# OASL promotes immune evasion in pancreatic ductal adenocarcinoma by enhancing autolysosome-mediated degradation of MHC-I

**DOI:** 10.7150/thno.103494

**Published:** 2025-01-13

**Authors:** Xin Xing, Xia-Qing Li, Shi-Qi Yin, Hong-Tai Ma, Shu-Yu Xiao, Aziguli Tulamaiti, Yan Yang, Shu-Heng Jiang, Li-Peng Hu, Zhi-Gang Zhang, Yan-Miao Huo, Dong-Xue Li, Xiao-Mei Yang, Xue-Li Zhang

**Affiliations:** 1 Shanghai University of Medicine & Health Sciences affiliated Sixth People's Hospital South Campus, Shanghai, China.; 2 Shanghai Fengxian District Central Hospital, School of Medicine, Anhui University of Science and Technology, Shanghai, China.; 3 State Key Laboratory of Systems Medicine for Cancer, Shanghai Cancer Institute, Ren Ji Hospital, School of Medicine, Shanghai Jiao Tong University, Shanghai, China.; 4 Department of Biliary-Pancreatic Surgery, Ren Ji Hospital, School of Medicine, Shanghai Jiao Tong University, Shanghai, China.

**Keywords:** PDAC, MHC-I, Autophagy, OASL, NBR1.

## Abstract

**Rationale:** Pancreatic ductal adenocarcinoma (PDAC) is a highly aggressive malignancy with a global prevalence and poor prognosis, largely due to immune escape mechanisms. However, the potential reasons for the decreased infiltration of cytotoxic T lymphocytes (CTLs) in PDAC remain inadequately understood. In this study, we aimed to elucidate the molecular mechanisms contributing to the low-CTLs infiltration in patients with PDAC.

**Methods:** Bioinformatic analyses were used to identify key factors associated with low-CTLs infiltration in PDAC and the role of oligoadenylate synthetase-like (OASL) was mainly focused in our study. Immunohistochemistry (IHC) was used to assess the relationship between the expression of OASL and the prognosis of patients. Western blotting, Flow cytometry, Co-immunoprecipitation and Immunofluorescence were applied to elucidate the molecular mechanism by which OASL mediates immune escape in PDAC. The orthotopic PDAC models were constructed to evaluate the effects of *OASL*-knockdown on CD8^+^ T cells infiltration and tumor growth *in vivo.*

**Results:** OASL was found to be significantly upregulated in PDAC and negatively correlated with the major histocompatibility complex class I (MHC-I) expression, which is associated with worse patient prognosis. Notably, *OASL-*knockdown leads to a significant increase in CD8^+^ T cell infiltration and slows tumor growth *in vivo*. Mechanistic studies revealed that *OASL* -knockdown restored the total and surface MHC-I level through impairing neighbor of BRCA1 gene 1 (NBR1)-mediated autophagy-lysosomal degradation of MHC-I.

**Conclusions:** Targeting OASL enhances the immune response in PDAC, providing a novel therapeutic strategy to improve outcomes in PDAC patients.

## Background

Pancreatic cancer, mainly pancreatic ductal adenocarcinoma (PDAC), is one of the most prevalent and lethal gastrointestinal malignancies in the world 1. It was reported that the 5-year survival rate is lower than 9% for patients with pancreatic cancer 2. Patients with PDAC who undergo the traditional surgical treatment and chemoradiotherapy often fail to achieve significant clinical benefits 3, 4. In recent years, with the development of immune therapies, immune checkpoint blockade (ICB) treatment and monoclonal antibodies against CTLA-4, PD-1, or PD-L1 extremely decelerated the progression of patients with cancer, such as lung cancer, melanoma, and bladder cancer 5-7. However, the majority of patients with PDAC fail to generate a good response to immunotherapy 8. PDAC represents an immunosuppressive "cold" tumor, making it difficult for infiltrated T-cells surrounding tumor cells to exert antitumor effects 9, 10. Therefore, the potential molecular mechanisms that regulate the growth of PDAC and T-cell immune response have not been fully explored yet. It is urgent to investigate the underlying mechanisms and their interactions in order to develop effective therapeutic strategies for PDAC treatment.

Major histocompatibility complex class I (MHC-I) plays a crucial role in antigen presentation and antitumor immunity. MHC-I is encoded by the human leukocyte antigen (HLA) class I genes HLA-A, HLA-B and HLA-C, which deliver intracellular protein fragments to CD8^+^T cells. Human leukocyte antigen-A (HLA-A) may directly affect the immune escape ability of tumor cells due to its high polymorphism and mutation frequency 11. MHC-I expressed on the surface of the most nucleated cells is a crucial link between neoantigen and cytotoxic T lymphocytes (CTLs) 12, 13. The loss of MHC-I on the surface of tumor cells is generally recognized as one of common immune escape mechanisms 14, 15. The downregulation or loss of MHC-I on tumor cells hampers the ability of CTLs to effectively recognize and target these tumor cells, allowing the tumor cells to evade immune surveillance by CTLs. Therefore, exploring the molecular mechanism responsible for the loss of MHC-I is an essential strategy, which can help us identify potential therapeutic targets to restore MHC-I expression and enhance the anti-tumor immune response mediated by CTLs.

The oligoadenylate synthetase-like (OASL) belongs to interferon-stimulated gene (ISG), which senses foreign nucleic acid and initiates anti-virus reaction 16-18. OASL is a member of the Oligoadenylate synthetases (OAS) family. When faced with virus infections, the body induces the production of OASL by activating IFN-I signaling. Then, OASL located in the cytoplasm enables to combine RIG-1 and simulate polyubiquitin, leading to enhanced sensitivity of RIG-1 and transmission of anti-virus signaling 19, 20. The previous studies have demonstrated that abnormal expression of OASL contributes to autoimmune diseases and anti-virus immune reactions 21. It is reported that OASL accelerates the proliferation, migration and invasion of cancers 22-24. However, the molecular mechanism of OASL in PDAC remains largely undetermined.

Autophagy, a protein degradation approach depending on the lysosome, degrades and recycles broken organelles and cell content. Five steps are involved in the process of autophagy, including the formation of isolation membranes, maturation of autophagosomes, closure of autophagosomes, fusion between autophagosomes and lysosomes, and lysosomal degradation 25. Physiologically, autophagy is a self-protection mechanism for inhibiting metabolic stress and oxidative damage. On the contrary, autophagy may lead to tumor and neurodegenerative disease when exposed to starvation, endoplasmic reticulum stress, oxidative stress and other stress conditions 26, 27. Previous studies have shown that autophagy plays a crucial role in PDAC growth and immune escape, and autophagy negatively regulates the expression of MHC-I in PDAC 28. During autophagy initiation, LC3 was exposed to carboxyl terminal Gly by cutting of autophagy-related protein 4 (ATG4), which is further activated by ATG7 (an E1-like enzyme) and transferred to ATG3 (an E2-like enzyme). Finally, LC3 was modified into LC3II participating in autophagy activation 29. LC3-II increases internalization of MHC-I molecules on the surface of dendritic cells (DCs), impeding activation of CD8^+^ cytotoxic T cells 30.

In this study, we found that OASL was highly expressed in PDAC compared with normal pancreas samples by multiple bioinformatics analysis. OASL facilitated immune evasion through decreasing expression of MHC-I on the surface of PDAC tumor cells and impeding activation of CD8^+^T cells. Mechanistically, OASL accelerated the loss of MHC-I through enhancing NBR1-mediated autophagy-lysosomal degradation of MHC-I in PDAC. These results suggest that OASL may be a potential target for treating immune evasion in PDAC.

## Results

### Identification of OASL as a crucial mediator of PDAC malignancy

**Figure 1:**
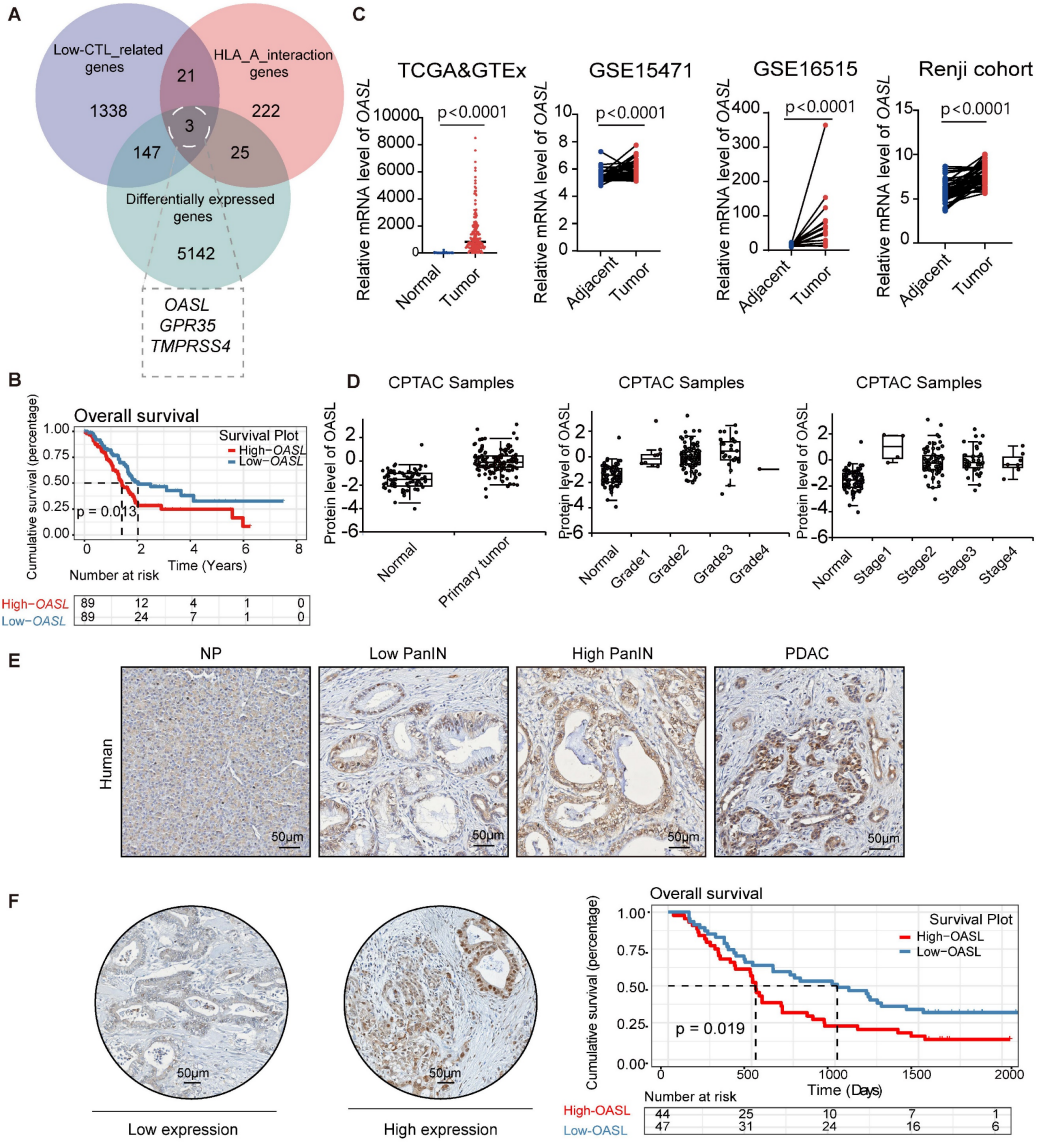
** The highly expressed OASL is positively correlated with the poor prognosis of patients with PDAC. (A)** Venn diagrams showing the common genes from MEturquoise Module genes from WGCNA, DEGs from TCGA and HLA-A interaction genes from the BioGRID database. **(B)** Kaplan-Meier curves (Log Rank test) of OS differences layered by the High-*OASL* and Low-*OASL* groups from TCGA data set. (C) Expression analyses of *OASL* in the pancreatic cancer and normal pancreas samples from the TCGA&GTEx (Adjacent = 167, Tumor = 179), GSE15471 (n = 39 per group), GSE16515 (n = 16 per group) and Renji cohort (n = 50 per group). **(D)** The differential expression of OASL at protein level in primary pancreatic tumor (n = 137) and normal pancreatic tissue (n = 74) from CPTAC Samples (p < 0.0001) (left). The protein expression level of OASL in different grades (Normal vs Grade1: p = 0.002), (Normal vs Grade2: p < 0.0001), (Normal vs Grade3: p < 0.0001) and (Normal vs Grade4: NA) (middle) and stages (Normal vs Stage1: p = 0.018), (Normal vs Stage2: p < 0.0001), (Normal vs Stage3: p < 0.0001) and (Normal vs Stage4: p = 0.004) (right) in pancreatic tumor from CPTAC Samples. **(E)** Representative immunohistochemical images of OASL expression in normal pancreas (NP), Low pancreatic intraepithelial neoplasia (PanIN), High PanIN and PDAC in human. **(F)** The immunohistochemical images and Kaplan-Meier survival analysis of PDAC patients with High-OASL and Low-OASL expression from Renji cohort (n = 91). Scale bar, 50 μm. Bars represent mean ± standard deviation. The p-value was calculated using a paired T-test in Figure 1C.

To identify the molecules associated with immune escape in pancreatic cancer, we calculated the abundance of immune cell infiltration in pancreatic cancer samples using the Microenvironment Cell Populations counter (MCP-counter) algorithm. MCP-counter was used to estimate the abundance of eight immune cells and two stromal cells populations 31, 32, 34, 35. The scarce infiltration of CTLs is an important factor that causes immune escape in PDAC. Therefore, the patients with pancreatic cancer were classified as the High-CTLs and Low-CTLs groups according to the median abundance of CTLs. The Weighted Gene Co-expression Network Analysis (WGCNA) is capable of clustering genes, constructing modules according to similarity of gene expression, and analyzing the associations between modules and biological traits 36. Thus, WGCNA was used for screening the most relevant modules with Low-CTLs in the pancreatic cancer samples from TCGA database (Figure S1A-C). The turquoise module (including 1509 genes) was positively associated with the Low-CTLs groups (cor = 0.22, p = 0.001) (Figure S1D). Additionally, 5317 differentially expressed genes (DEGs) were identified in pancreatic cancer samples and normal pancreas samples (Figure S1E). MHC-I plays a crucial role in tumor-recognition from cytotoxic CD8^+^ T cells through the presentation of tumor-antigens. 271 HLA-A interaction genes were found based on the BioGRID database. Then, the intersection of 5317 DEGs,1509 module genes in the most related with the Low-CTLs groups and 271 HLA-A interaction genes from BioGRID database identified 3 hub genes (*TMPRSS4*, *GPR35*, *OASL*) (Figure 1A). A Kaplan-Meier survival curve showed that the highly expressed* OASL* was positively associated with an unfavorable prognosis with PDAC patients (p = 0.013) (Figure 1B). However, Kaplan-Meier survival curves showed that there is no significant statistical difference between High-*TMPRSS4* and Low-*TMPRSS4* groups in pancreatic cancer (p = 0.12) (Figure S1F). The similar results were observed in High-*GPR35* and Low-*GPR35* groups in pancreatic cancer (p = 0.57) (Figure S1G). Therefore, *OASL* was identified as a potential molecular that may affect PDAC malignancy through regulating immune escape. To determine the expression pattern of OASL in PDAC, the adjacent and tumor samples from three databases (TCGA&GTEx, GSE16515, GSE15471) and Renji cohort were used for evaluating the mRNA expression level of* OASL*, illustrating that the abundance of *OASL* was greatly higher in PDAC samples compared with the adjacent pancreatic samples (Figure 1C). Simultaneously, the protein expression level of OASL was significantly higher in pancreatic tumor samples than normal samples according to the CPTAC database (Figure 1D). The expression level of OASL in pancreatic tumor samples with different clinical grades and stages was higher than normal pancreatic samples (Figure 1D). The immunohistochemical (IHC) staining analysis in PDAC samples demonstrated a progressive upregulation of OASL expression concomitant with disease advancement (Figure 1E). Furthermore, examination of a tissue microarray comprising PDAC specimens underscored that patients exhibiting elevated OASL expression were associated with a significantly worse prognosis (Figure 1F).

### OASL is negatively correlated with MHC-I in PDAC

**Figure 2:**
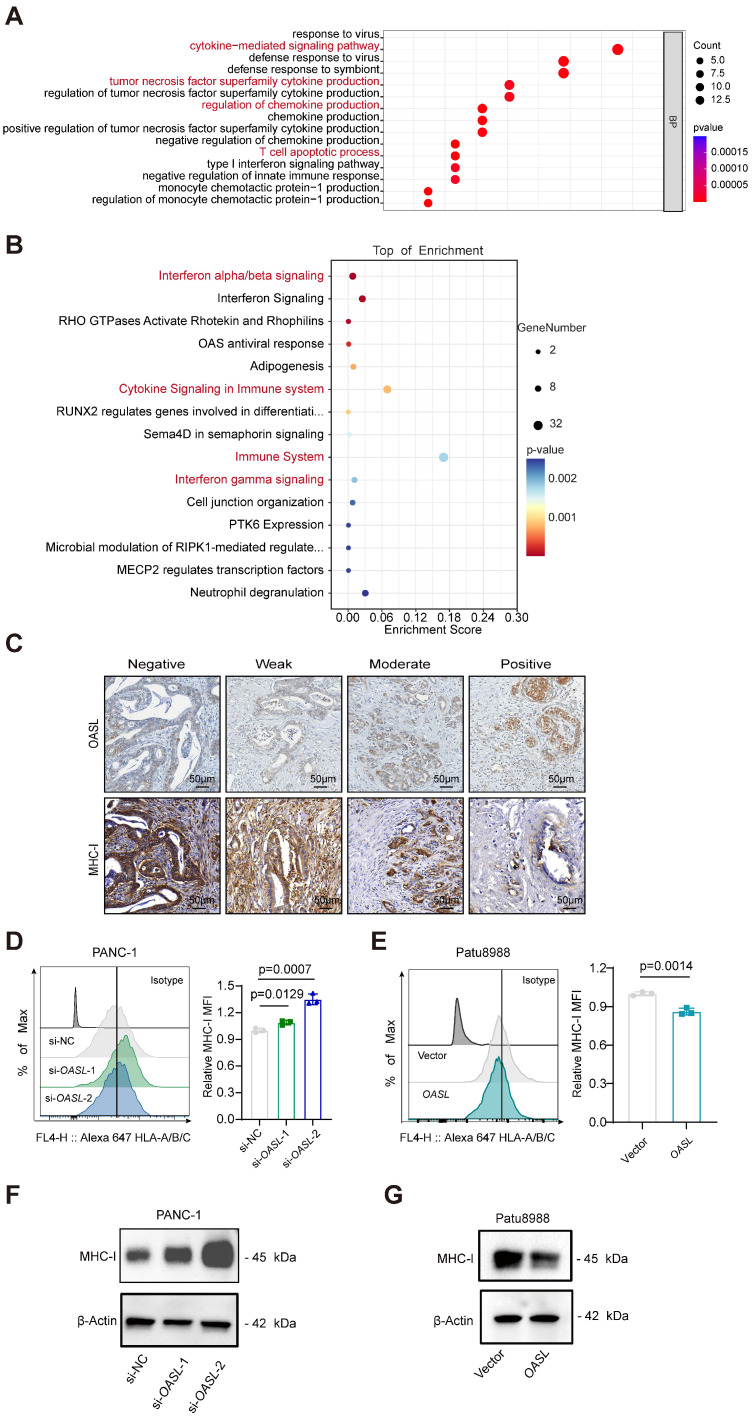
** OASL represses the expression of MHC-I. (A-B)** GO Biological Process analyses (A) and Reactome pathway analyses **(B)** of differentially expressed genes between High-*OASL* and Low-*OASL* groups from PAAD-TCGA database. **(C)** The immunohistochemical images for OASL and MHC-I in a tissue microarray from Renji cohort (n = 73) (Scale bar, 50 μm). (D-E) The expression of HLA-A/B/C on the surface of PDAC cell lines was measured by flow cytometry in PANC-1 cells with *OASL*-knockdown **(D)** and Patu8988 cells with *OASL-*overexpression **(E)** (n = 3, per group). (F-G) Western blot analysis of MHC-I in PANC-1 cells with *OASL*-knockdown **(F)** and Patu8988 cells with *OASL-*overexpression **(G)**. n = 3 biological replicates. Representative images were shown. Bars represent mean ± standard deviation. The p-value was calculated using an unpaired T-test in Figure 2D-E.

To determine whether OASL is involved in the immune infiltration process, GO biological process and Reactome pathway analyses of DEGs between High-*OASL* and Low-*OASL* groups were performed. Multiple immune-related pathways (such as cytokine-mediated signaling pathway, tumor necrosis factor superfamily cytokine production, Regulation of chemokine production, T cell apoptotic process and Interferon gamma signaling among others) were enriched (Figure 2A-B). The immunohistochemical staining of OASL and MHC-I was performed in normal pancreas and pancreatic cancer tissues of humans. A tissue microarray of PDAC specimens demonstrated that the expression level of MHC-I gradually decreased with the increasing expression of OASL (Figure 2C and S1H). The immuno-histochemical images of OASL expression and MHC-I expression indicated that increased OASL expression accompanies with decreased MHC-I expression during PDAC progression (Figure S1I). To further investigate whether OASL affects the expression of MHC-I in PDAC cells, we examined the expression levels of OASL in various PDAC cell lines by qPCR and Western blot (Figure S2A-B). Two relatively high-OASL expression cell lines were selected for knockdown of *OASL* (PANC-1 cells and MIA PaCa-2 cells), and two relatively low-OASL expression cell lines for overexpression of *OASL* (AsPC-1 cells and Patu8988 cells). PANC-1 cells and MIA PaCa-2 cells were transfected with small interfering RNA and the efficiency of *OASL*-knockdown was validated by qPCR and Western blot analysis, respectively (Figure S2C-F). Similarly, overexpression of *OASL* was transfected in AsPC-1 cells and Patu8988 cells and the efficiency of overexpression of *OASL* was greatly significant increased based on qPCR and Western blot analysis (Figure S2G-J). To explore the potential relationship of OASL and MHC-I, the expression of MHC-I on the surface of tumor cells was measured by flow cytometry after the knockdown of *OASL* and overexpression of *OASL*, respectively. The expression of MHC-I on the surface of tumor cells was increased by *OASL*-knockdown (Figure 2D and Figure S2K), while overexpression of *OASL* can significantly reduce the expression of MHC-I on the surface of tumor cells (Figure 2E and Figure S2L). Furthermore, the total protein level of MHC-I was enhanced by Western blot in PANC-1 cells and MIA PaCa-2 cells with *OASL*-knockdown, as detected by Western blot (Figure 2F and Figure S2M), while it was reduced in cell lines with overexpression of *OASL* (Figure 2G and Figure S2N). The results indicate that OASL had a negative regulation of MHC-I in PDAC.

### OASL accelerates the degradation of MHC-I which is dependent on the lysosomal pathway

**Figure 3:**
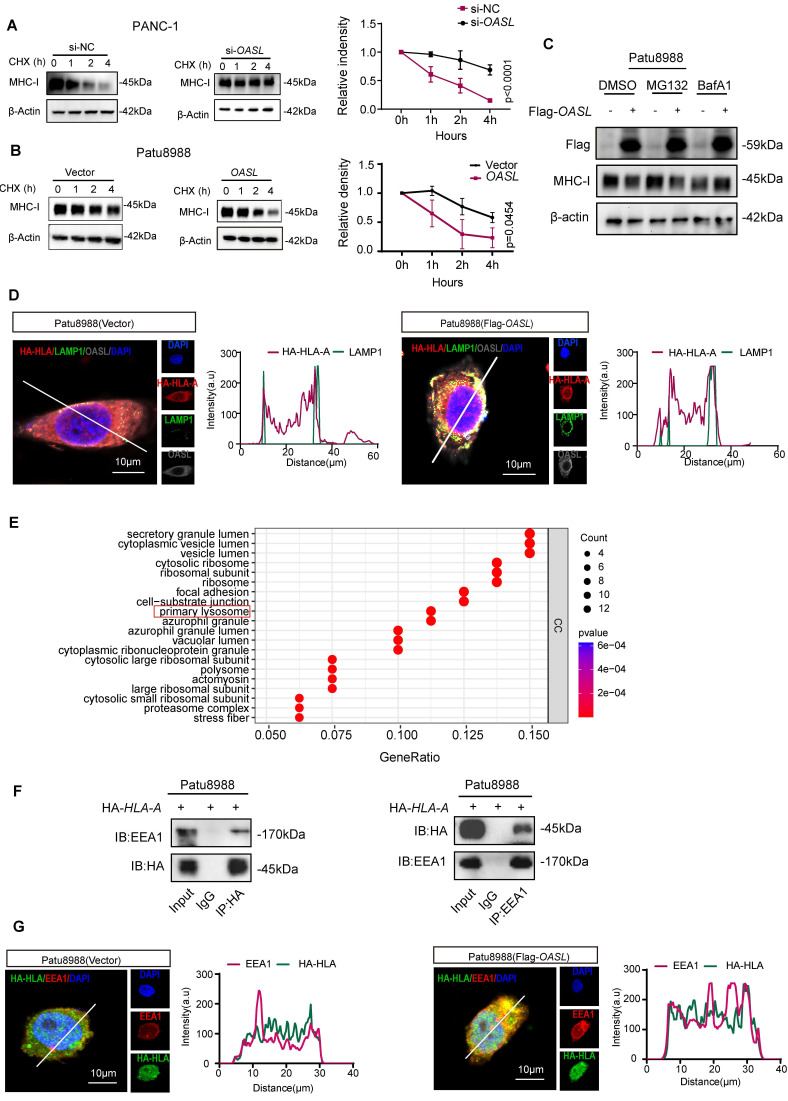
** OASL promotes the degradation of MHC-I by autolysosome. (A-B)** The half-life of MHC-I was evaluated in PANC-1 cells with *OASL*-knockdown (A) (p < 0.0001) and Patu8988 cells with overexpression of *OASL* **(B)** (p = 0.0454) treated with CHX (20μM) at the specific time point by Western blot (left). The protein half-life curves were obtained by quantifying relative intensities (right). n = 3 biological replicates. Representative images were shown. **(C)** Patu8988 cells with overexpression of *OASL* were treated with dimethyl sulfoxide (DMSO), proteasome inhibitor MG132 (20μM) and lysosomal inhibitor BafA1 (20μM) for 4 h, and the expression of MHC-I was evaluated by Western blot. n = 3 biological replicates. Representative images were shown. **(D)** Patu8988 cells stably expressing HA-*HLA-A* were transfected with Flag-*OASL* (Pearson's R value = 0.58) and Vector (Pearson's R value = 0.44) and were stained for HA-HLA-A (red), LAMP1 (green) and OASL (gray). The cell nucleus (blue) was stained with DAPI. The colocalization was visualized by confocal microscopy. Representative immunofluorescence images were shown (Scale bar, 10 μm) (left). Image analysis of immunofluorescence staining intensity across the line was shown in the right panel. **(E)** GO analysis based on Co-IP and mass spectrum (MS). **(F)** The relationship between HLA-A and EEA1 was analyzed by Co-IP analysis. Patu8988 cells were stably transfected with HA-HLA. The whole-cells were immunoprecipitated with anti-HA beads and followed by Western blot with antibodies against EEA1 (left). The whole-cells were immunoprecipitated with anti-EEA1 beads and followed by Western blot with antibodies against the HA (right). n = 3 biological replicates. Representative images were shown. **(G)** Patu8988 cells stably expressing HA-*HLA-A* were transfected with Flag-*OASL* and Vector and were stained for EEA1 (red), HA-HLA (green). The cell nucleus (blue) was stained with DAPI. The colocalization of EEA1 and HA-HLA-A was visualized by confocal microscopy. Representative immunofluorescence images were shown (Scale bar, 10 μm) (left). Image analysis of immunofluorescence staining intensity across the line was shown in the right panel. Bars represent mean ± standard deviation. The p-value was calculated using Two-way ANOVA in Figure 3A-B.

To explore how MHC-I expression was impaired by OASL, the half-life of MHC-I was measured by blocking protein synthesis using cycloheximide (CHX). MHC-I was degraded more slowly in PANC-1 cells and MIA PaCa-2 cells (Figure 3A and Figure S3A) with *OASL*-knockdown than in control cells. On the contrary, the protein level of MHC-I decreased more rapidly in Patu8988 cells and AsPC-1 cells (Figure 3B and Figure S3B) with Flag-*OASL* compared with the Vector control cells. This demonstrated that OASL impaired the protein stability of MHC-I in PDAC. Protein degradation occurs mainly in lysosomes and/or proteasomes 37. Therefore, we treated Patu8988 cells and AsPC-1 cells with V-ATPase inhibitor bafilomycin A1 (BafA1) to inhibit lysosome function or MG132 to block the function of proteasome. In Patu8988 cells and AsPC-1 cells transfected with Vector or Flag-*OASL*, the expression of MHC-I was significantly decreased in the overexpression of *OASL* groups compared with control groups, regardless of whether treatment with MG132 was administered (Figure 3C and Figure S3C). This indicated that the regulation of MHC-I by OASL was not dependent on the proteasome pathway. When Patu8988 cells and AsPC-1 cells transfected with Vector or Flag-*OASL* were treated with BafA1, the expression of MHC-I was restored in the *OASL*-overexpression cells (Figure 3C and Figure S3C). Thus, it was suggested that OASL downregulated MHC-I through the lysosomal pathway. In addition, HA-HLA showed stronger colocalization with LAMP1 (a lysosome marker) in Patu8988 cells and AsPC-1 cells with Flag-*OASL* compared with Vector (Figure 3D and Figure S3D). Furthermore, the enrichment analysis from Co-IP and mass spectrometry assays were performed and showed that OASL was related to primary lysosome (Figure 3E). The previous research reported that OASL participate in the endosomal sorting process 38. Early endosome antigen 1 (EEA1) is a major marker of the early endosome stage. The colocalization of OASL and EEA1 was observed in Patu8988 cells and AsPC-1 cells (Figure S3E-F). The interaction of HLA-A and EEA1 was further verified by Co-IP assay in Patu8988 cells and AsPC-1 cells (Figure 3F and Figure S3G). Similarly, HLA-A and EEA1 were strongly colocalized in Patu8988 cells and AsPC-1 cells with Flag-*OASL* compared with Vector (Figure 3G and Figure S3H). OASL promotes the entry of HLA-A into early endosomes and provides conditions for degradation in lysosomes. Collectively, our data show that OASL promotes the degradation of MHC-I in the lysosome.

### OASL mediates MHC-Ι degradation through autophagy

**Figure 4:**
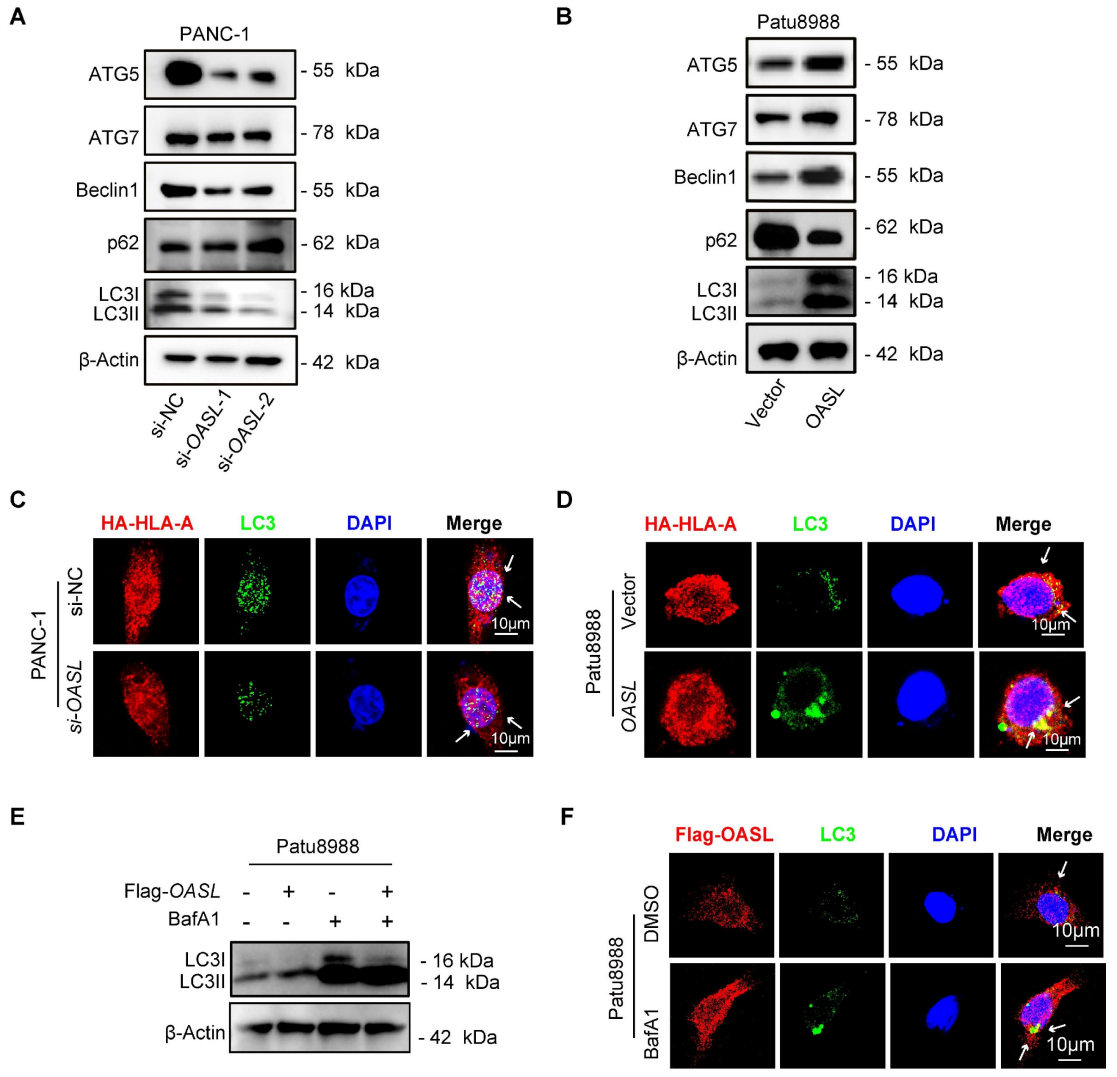
**OASL participates in the autophagy**. **(A-B)** The expression levels of ATG5, ATG7, Beclin1, p62 and LC3 (LC3-I and -II) were detected by Western blot analysis in PANC-1 cells with *OASL*-knockdown **(A)** and Patu8988 cells with *OASL*-overexpression **(B)**. n = 3 biological replicates. Representative images were shown. (C-D) PANC-1 cells with *OASL*-knockdown **(C)** and Patu8988 cells with *OASL*-overexpression **(D)** stably expressing HA-*HLA-A* were stained for HLA-A-HA (red) and LC3 (green). The cell nucleus (blue) was stained with DAPI. The colocalization was visualized by confocal microscopy. Representative immunofluorescence images were shown. (Scale bar, 10 μm). **(E)** Western blot analysis of LC3 (LC3-I and -II) levels in Patu8988 cells expressing Flag-*OASL* upon treatment with or without BafA1. n = 3 biological replicates. Representative images were shown. **(F)** Patu8988 cells with Flag*-OASL* were treated with DMSO (4 h) or BafA1 (4 h) and stained for Flag-OASL (red) and LC3 (green). The cell nucleus (blue) was stained with DAPI. The colocalization was visualized by confocal microscopy. Representative immunofluorescence images were shown. (Scale bar, 10 μm).

It has been reported that lysosomes are involved in the degradation of autophagy cargo 39. Thus, to determine whether autophagy participates in MHC-I degradation, we first examined the relationship between OASL and autophagy. Western blot analysis demonstrated that the expression of ATG5, ATG7, Beclin1 and LC3 (autophagosome markers) was decreased by* OASL*-knockdown in PANC-1 cells and MIA PaCa-2 cells (Figure 4A and Figure S4A), while overexpression of *OASL* significantly increased the expression of ATG5, ATG7, Beclin1 and LC3 in Patu8988 cells and AsPC-1 cells (Figure 4B and Figure S4B). p62, a scaffold ubiquitin-binding protein that interacts with ubiquitinated protein aggregates, was degraded by autophagy 40. The inhibition of autophagy facilitates p62 accumulation, whereas the activation of autophagy leads to decreased p62 levels 41. The expression level of p62 was increased in PANC-1 cells and MIA PaCa-2 upon *OASL-*knockdown (Figure 4A and Figure S4A). On the contrary, a decrease in the level of p62 was observed in Patu8988 cells and AsPC-1 cells with *OASL*-overexpression (Figure 4B and Figure S4B), suggesting that OASL induced autophagy in PDAC. Then, we examined the colocalization between HLA-A and autophagosomes within Patu8988 cells and AsPC-1 cells. In PANC-1 and MIA PaCa-2 cells with *OASL*-knockdown, a significant decrease in the proportion of HLA-A co-localizing with LC3-labeled autophagosomes was observed compared to control cells (Figure 4C and Figure S4C). Conversely, a notable increase in the proportion of HLA-A co-localizing with LC3-labeled autophagosomes was detected in Flag-*OASL*-expressing Patu8988 cells and AsPC-1 cells compared to the control cells (Figure 4D and Figure S4D). Western blot analysis showed that OASL significantly increased LC3 levels in Patu8988 cells and AsPC-1 cells expressing Flag-*OASL* treated with BafA1, which blocked the degradation of autophagosomes (Figure 4E and Figure S4E). An increasing colocalization of OASL and LC3 (an autophagosome marker) was observed in Flag-*OASL* Patu8988 cells and AsPC-1 cells after BafA1 treatment (Figure 4F and Figure S4F). Chloroquine (CQ), a clinically available drug, was used to inhibit autophagy. When Patu8988 cells and AsPC-1 cells transfected with Vector or Flag-*OASL* were treated with CQ, the expression of MHC-I was restored in the *OASL*-overexpression cells (Figure S5A-B). An increasing colocalization of OASL and LC3 was observed in Flag-*OASL* Patu8988 cells and AsPC-1 cells after CQ treatment (Figure S5C-D). Western blot analysis showed that OASL significantly increased LC3 levels in Patu8988 cells and AsPC-1 cells expressing Flag-*OASL* treated with CQ (Figure S5E-F). Together, these results suggest that OASL might hijack MHC-I into the autophagy initiation pathway to induce MHC-I degradation.

### OASL promotes MHC-I trafficking to lysosomes via NBR1

**Figure 5:**
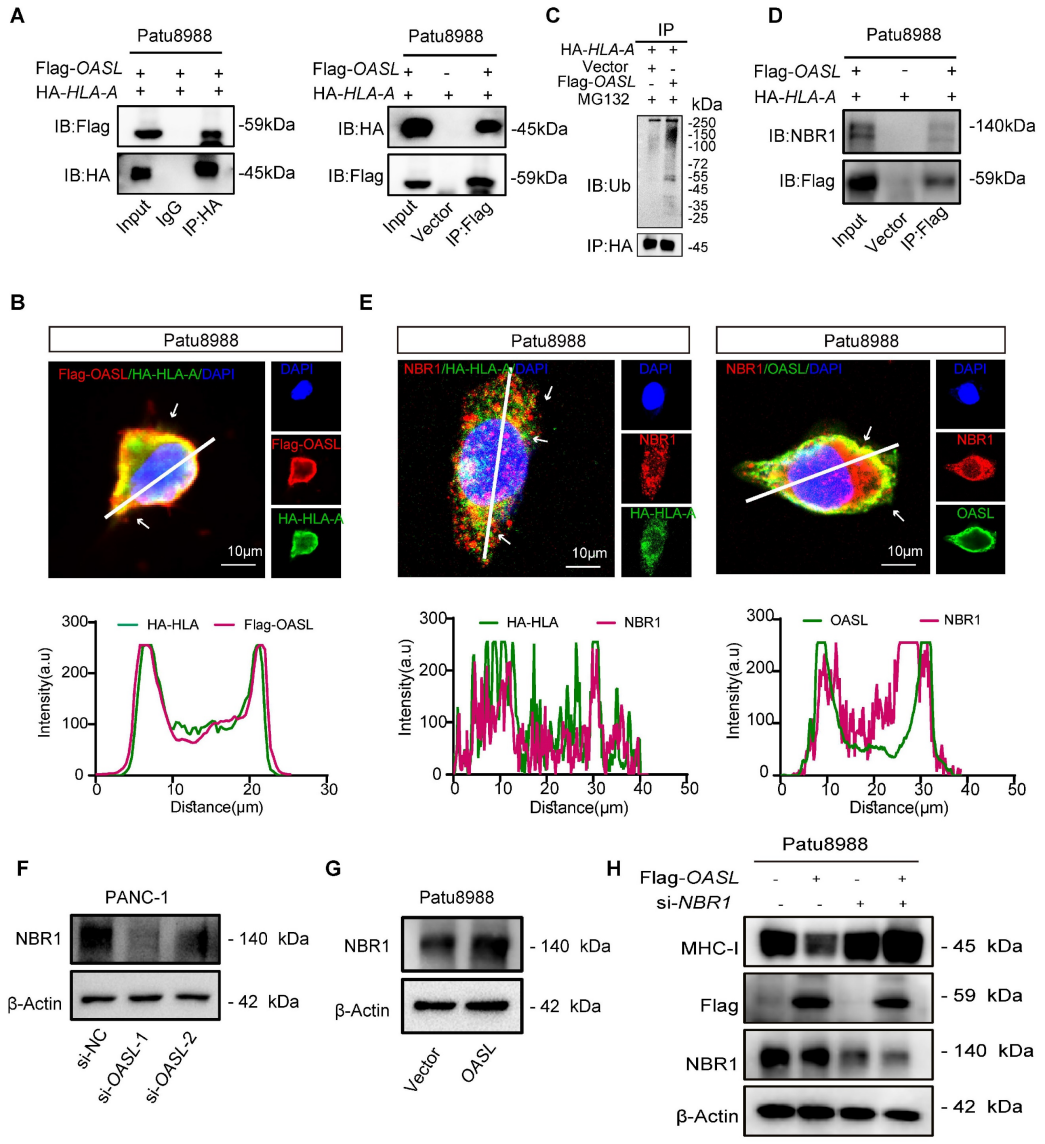
** OASL promotes MHC-I trafficking to lysosomes via NBR1. (A)** The interaction of OASL and HLA-A was analyzed by Co-IP analysis. Flag-*OASL* was transfected in Patu8988 cells stably expressing with HA-*HLA* (left). The whole-cells were immunoprecipitated with anti-HA beads and followed by Western blot with antibodies against the Flag. Vector and Flag-*OASL* were transfected in Patu8988 cells with HA-*HLA*. The whole-cells were immunoprecipitated with anti-Flag beads and followed by Western blot with antibodies against the HA (right). n = 3 biological replicates. Representative images were shown. **(B)** Patu8988 cells stably expressing HA-*HLA-A* were transfected with Flag-*OASL* and were stained for HA-HLA-A (green) and Flag-OASL (red). The cell nucleus (blue) was stained with DAPI. The colocalization was visualized by confocal microscopy. Representative immunofluorescence images were shown (Scale bar, 10 μm) (up). Image analysis of immunofluorescenct staining intensity across the line was shown in the right panel (bottom). **(C)** Vector and Flag-*OASL* were transfected in Patu8988 cells with HA-*HLA* and treated with MG132 (20μM) for 4 h. Cellular extracts were immunoprecipitated with anti-HA and followed by Western blot with anti-ubiquitin (Ub) antibody. n = 3 biological replicates. Representative images were shown. **(D)** Patu8988 cells expressing Flag-*OASL* and HA-*HLA-A* were analyzed by Co-IP and Western blot. The whole-cells were immunoprecipitated with anti-Flag beads and followed by Western blot with antibodies against the NBR1. n = 3 biological replicates. Representative images were shown. **(E)** Patu8988 cells stably expressing HA-*HLA-A* were transfected with Flag-*OASL* and were stained for NBR1 (red) and HA-HLA-A (green). The cell nucleus (blue) was stained with DAPI. The colocalization was visualized by confocal microscopy. Representative immunofluorescence images were shown. Scale bar, 10 μm) (left). Patu8988 cells stably expressing HA-*HLA-A* were transfected with Flag-*OASL* and were stained for NBR1 (red) and OASL (green). The cell nucleus (blue) was stained with DAPI. The colocalization was visualized by confocal microscopy. Representative immunofluorescence images were shown. Scale bar, 5μm) (up). Image analysis of immunofluorescenct staining intensity across the line was shown in the down panel (down). **(F-G)** The expression of NBR1 was measured by Western blot in PANC-1 cells with *OASL-*knockdown **(F)** and Patu8988 cells expressing Flag-*OASL* **(G)**. n = 3 biological replicates. Representative images were shown. **(H)** Western blot analysis of MHC-I expression levels in Patu8988 cells expressing Flag-*OASL* with or without *NBR1-*knockdown. n = 3 biological replicates. Representative images were shown.

To further uncover the mechanism of MHC-I degradation promoted by OASL, immuno-precipitation coupled with mass spectrometry (IP-MS) experiments were performed to identify proteins interacted with OASL from cellular extracts of Patu8988 cells with Flag-*OASL*. The mass spectrometry results indicated that HLA-A was coprecipitated with Flag-*OASL* (Figure S5G). The interaction of OASL and HLA-A was further verified by Co-IP assay in Patu8988 cells and AsPC-1 cells (Figure 5A and Figure S5H). Furthermore, we found that OASL colocalized with HLA-A in Patu8988 cells and AsPC-1 cells (Figure 5B and Figure S5I). Previous studies have found that OASL contains two tandem ubiquitin-like domains that are functionally similar to K63 ubiquitin-like modifications 38. The Co-IP assay indicated that the ubiquitination level of HLA-A was higher in Patu8988 cells with expressing Flag-*OASL* than control cells (Figure 5C). This result showed that OASL promoted the ubiquitination and degradation of MHC-I in PDAC. Additionally, a neighbor of BRCA1 gene 1 (NBR1) is generally recognized as a common autophagy cargo receptor that selectively degrades proteins by targeting ubiquitylated substrates 28. Therefore, we reasoned that the MHC-I deficiency was largely caused by NBR1-mediated autophagy-lysosomal degradation. To confirm the hypothesis, we initially examined whether OASL can bind to HLA-A and NBR1 using immunoprecipitation assays. In accordance with the hypothesis, we verified the interaction of OASL with HLA-A and NBR1 in Patu8988 cells by Co-IP assay (Figure 5D). Additionally, the immunofluorescence staining exhibited physical colocalization of HLA-A and NBR1, OASL and NBR1 in Patu8988 cells (Figure 5E). Then, we found a significantly positive correlation between *OASL* and *NBR1* through the GEPIA2 database (Figure S6A), which was further confirmed by Western blot assay in PDAC (Figure 5F-G and Figure S6B-C). To determine whether OASL drives the loss of MHC-I through NBR1, we detected the expression of MHC-I in *OASL*-overexpressed AsPC-1 cells and Patu8988 cells with *NBR1*-knockdown. The efficiency of *NBR1-*knockdown was validated by qPCR and Western blot analysis in Patu8988 cells and AsPC-1 cells (Figure S6D-G). Subsequently, a significant increase of MHC-I total protein levels was observed based on *NBR1*-knockdown and the expression levels of MHC-I were also fully rescued in Flag-*OASL* overexpression Patu8988 cells and AsPC-1 cells by *NBR1*-knockdown (Figure 5H and Figure S6H). Collectively, our results demonstrated OASL downregulates MHC-I through NBR1-mediated autophagy-lysosomal degradation.

### Knockdown of OASL inhibits the growth and autophagy of PDAC and enhances immune response

**Figure 6:**
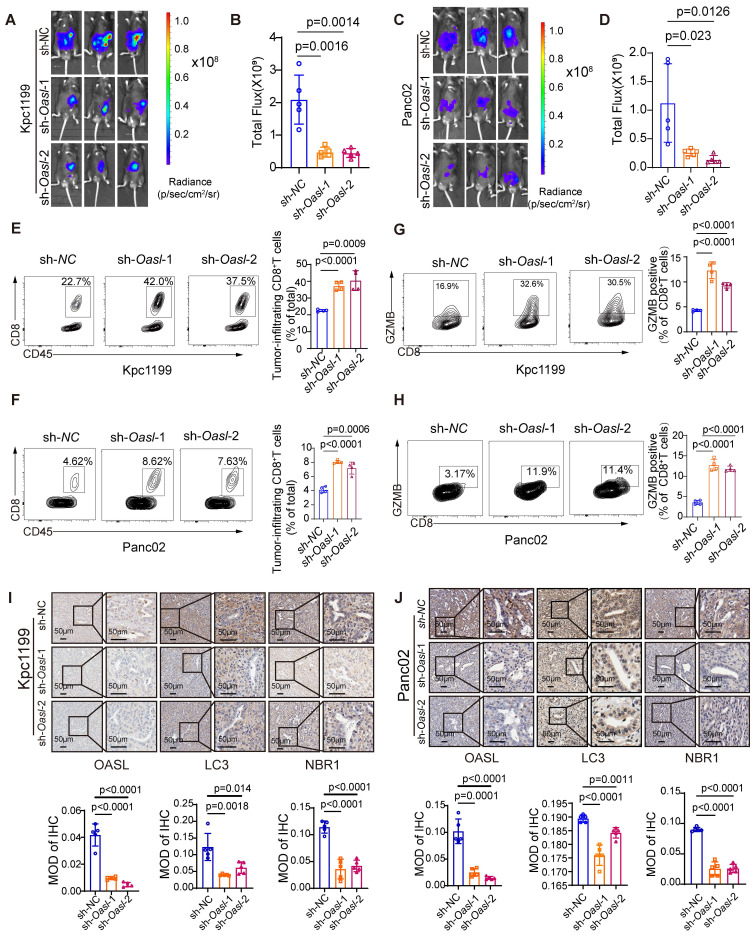
***OASL*-knockdown inhibits tumor growth and promotes CD8^+^ T infiltration. (A-D)** Kpc1199-luc cells and Panc02-luc cells with stable depletion of *Oasl* were orthotopically injected to pancreas in C57BL/6J mice, and bioluminescence images and quantitative results were shown (n = 5, per group).** (E-F)** The tumor-infiltrating CD8^+^T cells were analyzed by flow cytometry in immune microenvironment from mice that were orthotopically injected with Kpc1199 cells and Panc02 cells bearing sh-NC, sh-*Oasl*-1 and sh-*Oasl*-2 (n = 4, per group). **(G-H)** The amount of GZMB was analyzed by flow cytometry in the immune microenvironment of tumors that were orthotopically injected with Kpc1199 cells and Panc02 cells bearing sh-NC, sh-*Oasl*-1, sh-*Oasl*-2 (n = 4, per group). **(I-J)** The immunohistochemical staining of OASL, LC3 and NBR1 in pancreatic tumors derived from Kpc1199 and Panc02 cells with *Oasl*-knockdown. Scale bar, 50 μm. The mean optical density (MOD) was used for evaluating the protein expression by image J (n = 5). Bars represent mean ± standard deviation. The p-value was calculated using an unpaired T-test in Figure 6B, 6D and 6E-J.

To reveal the roles of OASL in the progression of PDAC *in vivo*, we successfully established *Oasl* knockdown cell lines in Kpc1199 (The *Kras^G12D/+^/Trp53^R172H/+^/Pdx-1-*Cre (KPC) mouse-derived syngeneic PDAC cell line) and Panc02 (murine PDAC cell line). The previous research had demonstrated that* Oasl* was classified into two *Oasl* genes in mice, *Oasl1* and *Oa*sl2. It has been reported that m*Oasl1* has greater (70%) similarity to h*OASL* compared with m*Oasl2* (48%) in amino acid sequence 42. In addition, NTase activity is lost in h*OASL* and m*Oasl1*, but m*Oasl2* has NTase activity 43. The mouse *Oasl2* and human *OASL* are functionally similar. It is reported that human *OASL* and its mouse ortholog, *Oasl2*, enhance RNA-sensor RIG-I-mediated type I interferon (IFN) induction and inhibit RNA virus replication 44. Human *OASL* and mouse* Oasl2* inhibit DNA virus infection by inhibiting cGAS-mediated IFN induction 45. Both studies demonstrated that their conserved mechanisms in immune regulation. These functional similarities make mouse *Oasl2* a suitable model for investigating the biological roles of human* OASL*, particularly in understanding immune responses. Therefore, mouse* Oasl2* was selected (*Oasl2* refer to *Oasl*) in order to investigate the function of *OASL* in our study. Firstly, the knockdown efficiency of *Oasl* was validated by qPCR and Western blot analysis (Figure S7A-D). Simultaneously, the expression of H-2K^b^ (murine MHC-I) on the surface of murine PDAC cells (Kpc1199 and Panc02) bearing NC, sh-*Oasl-1* and sh-*Oasl-2* was measured by flow cytometry, and the results showed that the expression of H-2K^b^ on the surface of murine PDAC cells was significantly increased after *Oasl*-knockdown (Figure S7E-F). Then, Kpc1199 and Panc02 cells bearing NC, sh-*Oasl-*1 and sh-*Oasl-*2 were respectively injected into the pancreas of C57BL/6J mice to construct orthotopic PDAC models. The bioluminescence imaging indicated that pancreas orthotopic tumors grew slower in sh-*Oasl-*1* and* sh-*Oasl-*2 groups than sh-NC groups (Figure 6A-D). The tumor size was smaller, and the weight was lighter in sh-*Oasl-1 and* sh-*Oasl-2* groups than sh-NC groups (Figure S8A-B). The proportion of infiltrated immune cells and immune components had been largely changed in sh-*Oasl-1 and* sh-*Oasl-2* groups, as compared to NC group. Flow cytometry revealed that the percentage of CD8^+^ T cells was significantly higher in sh-*Oasl-*1* and* sh-*Oasl-*2 groups than that in the NC group (Figure 6E-F). Besides, this release of GZMB, IFN-γ and TNF-α was increased in the infiltrated CD8^+^T cells from sh-*Oasl-1 and* sh-*Oasl-*2 tumors than that from the NC group (Figure 6G-H and Figure S8C-F). The immunohistochemical staining also demonstrated that the Ki67^+^ cells were less in *Oasl*-knockdown tumors than the control counterparts (Figure S9A-B). The immuno-histochemical staining also demonstrated the increased amount of CD8 and GZMB in *Oasl*-knockdown tumors compared to the control counterparts (Figure S9C-F). Furthermore, a tissue microarray of PDAC specimens demonstrated that the expression level of CD8 and GZMB was gradually decreased accompanied with the increasing expression of OASL (Figure S9G).

To determine whether OASL promotes the progression of PDAC through autophagy *in vivo,* we examined the expression of LC3 and NBR1 in mouse PDAC samples. The immunohistochemical analysis displayed that the tissues from the sh-*oasl*-1 and sh-*oasl*-2 groups showed a decrease in the expression of LC3 and NBR1 as compared to the tissues from the control group (Figure 6I-J). These results confirm that OASL promotes autophagy *in vivo* in PDAC. These results revealed that *OASL-*knockdown inhibited tumor growth and promoted immune activation in PDAC. All in all, inhibition of OASL strongly blunts PDAC progression by inhibiting autolysosome-mediated degradation of MHC-I.

## Methods

### Data mining

The RNA-seq data for transcriptome profiling of 178 pancreatic cancer patients were obtained from TCGA database. Meanwhile, the RNA-seq data of 167 normal pancreatic samples were downloaded from the GTEx database in the UCSC Xena database (http://xena.ucsc.edu). The protein expression of OASL was analyzed on the Clinical Proteomics Consortium for Cancer Analysis (CPTAC) dataset by UALCAN website (https://ualcan.path.uab.edu/analysis-prot.html). The relationship between OASL and NBR1 in pancreatic cancer was analyzed using the Gene Expression Profiling Interactive Analysis (GEPIA2) database (http://gepia.cancerpku.cn/index.html).

The different immune cell scores of pancreatic cancer patients from the TCGA databases were calculated using the MCP-counter algorithm provided by TIMER 2.0 (http://timer.cistrome.org/) 31, 32. Then, the patients with pancreatic cancer were classified as the High-CTLs and Low-CTLs groups according to the median abundance of CTLs. The co-expression network analysis was performed using the Weighted Gene Correlation Network Analysis (WGCNA) R-package. The Low-CTLs related genes were filtered from the most significant modules related to Low-CTLs groups. To screen for DEGs between pancreatic cancer samples and normal pancreas samples in the TCGA database, the analysis of differential gene expression was performed using the Bioconductor limma package of the R software. The genes that interact with HLA-A (Table S1) were downloaded from interaction database analysis from the BioGRID database (https://thebiogrid.org/).

### Clinical samples

The tissue samples from the Department of Hepatobiliary Surgery, Renji Hospital affiliated to Shanghai Jiao Tong University, were collected for the construction of the tissue microarray. The informed consent was obtained from all patients. All procedures are in accordance with the regulations of China Ethics Review Committee.

### Cell culture

Human pancreatic adenocarcinoma cells (AsPC-1, PANC-1, MIA PaCa-2, Patu8988, CFPAC-1, SW1990, BxPC-3), and human normal pancreatic duct epithelial cells (HPDE) were all preserved in Shanghai Cancer Institute, Shanghai Jiao Tong University. AsPC-1, BxPC-3 and CFPAC-1 cells were kept in RPMI 1640 with 10% fetal bovine serum (FBS) (Gibco), PANC-1, Patu8988, MIA PaCa-2, SW1990 and HPDE cells were cultured by Dulbecco's modified Eagle medium (DMEM) with 10% FBS (Gibco). The mouse pancreatic cancer cell lines KPC1199 and Panc02 cells were kept in DMEM with 10% FBS. Passage was performed with 80-90% cells density according to rate of cell growth. All cells were incubated in the specific incubator (5% CO_2_ at 37 °C).

### Transient transfection

The short interfering RNAs (siRNAs) targeting human *OASL* were purchased from Biotend Biotechnology (Shanghai, China). Sequences of siRNA are as follows,

si-*OASL*-1: 5'-GCAGAGAAAUUUCGUGAAACATT-3';

si-*OASL*-2: 5'-GGUUCUCAGGAGCACCAGAGATT-3';

si-*NBR1*-1: 5'*-*GCAUGAUCAGCUCAAGCAATT-3';

si-*NBR1*-2: 5'-UUGCUUGAGCUGAUCAUGCTT-3'.

pcDNA3.1(+)-OASL-3xFlag and the negative control plasmid pcDNA3.1(+)-MCS-3xFlag were purchased from Obio Technology (Shanghai, China). Cells were transfected using JetPRIME (Polyplus transfection,101000046) according to the manufacturer's instructions.

### Construction of stable cell lines

pLenti-U6-shRNA (*Oasl2*)-CBh-3xFLAG-Luc2-tCMV-mNeonGreen-F2A-Puro-WPRE Lentiviral particles targeting the mouse *Oasl2* gene were purchased from Obio Technology (Shanghai, China). Lentiviral particles of human *HLA-A2* (XM_041680767.1) were purchased from Bio-lifespan (Shanghai, China). The mixture of Lentiviral particles and polybrene was added to the 6-well plates for transfection when the cells that were seeded into 6-well plates had reached about 50%. The cells that were approximately infected for 72 h were treated with 2 µg/mL puromycin (Yeasen, 60210ES60) for 7 days to obtain stable *Oasl2*-knockdown or stable *HLA-A2* overexpressing cells.

### RNA extraction and real-time quantitative polymerase chain reaction (RT-qPCR)

The Total RNA was extracted using TRIzol reagent (Takara). The first-strand cDNA was reverse-transcribed by the All-in-One First-Strand Synthesis Master Mix Reagent Kit (ShareBio, Shanghai, China). qRT-PCR was performed using SYBR green qPCR Premix (ShareBio, Shanghai, China) on a 7500 Real-time PCR system (Applied Biosystems, Inc. USA). The expression levels of target genes were calculated by comparing to the expression of the reference gene 18s, and quantification was performed using the 2-ΔΔctmethod. The related experiments were performed in triplicate. The sequences of primers were shown in Table S2.

### Reagents

Detailed information on all reagents used in this article is listed in Table S3.

### RNA-seq analysis

Total RNA was extracted from MIA-PaCa-2 cells in the NC and *OASL*-knockdown groups using Trizol (Takara) according to the manufacturer's protocol. The transcriptome sequencing and analysis were conducted by OE Biotech Co., Ltd. (Shanghai, China).

The raw RNA-seq data have been deposited in NCBI SRA (https://www.ncbi.nlm.nih.gov/sra) under the accession number PRJNA1144669.

### Animal studies

The aged 6-8-week-old wild-type C57BL/6J mice were used to construct orthotopic PDAC models. The feeding process of mice is in accordance with the Shanghai Jiao Tong University Animal Care Commission. When murine PDAC cells (Kpc1199 and Panc02) reached 70-80% confluence, these cultured cells were washed and resuspended in PBS. The tumor cells were suspended in 25 μL PBS. PBS was injected into the pancreas of C57BL/6 mice. Tumor size was measured every 7 days using the living image system.

### Flow cytometry

The cultured cells are digested by trypsin and washed with PBS. The antibody (human HLA-ABC, mouse H-2Kb) diluted by FACS buffer (2% FBS in PBS) was incubated with cells at 4 °C for 30 min away from light. Add 1000 µL FACS buffer to terminate stain, followed by centrifugation at 4°C at 5000 rpm for 5 min. The cells were resuspended with a 300 uL FACS buffer and transferred into the flow tube. Flow cytometry analysis was conducted using a BD Fortessa FACS with FlowJo software v10.8. For sample preparation *in vivo*, mouse tumor tissue was cut into small pieces and digested with 1 mg/ml collagenase A (Sigma) and 1× DNase I (Sigma) for 20 min. Digestion was terminated with 3-5 mL medium containing 5% FBS. The liquid with fragments is filtered with 70 μm nylon filter and washed with PBS. Lymphocytes that were isolated with Ficoll gradient were stimulated with leukocyte activation cocktail (BD, 550583) for 4 h and then neutralized with 500 μL FACS buffer. The antibodies against CD45, S780, and CD8 were applied for cell surface staining. Then, cells were fixed and permeabilized with the Fix/Perm kit (BD Biosciences, 51-2090KZ) and stained with antibodies against GZMB, TNF-α, and IFN-γ. The stained cells were analyzed by flow cytometry (LSRFortessa, Becton Dickinson). The detailed antibodies information was found in Table S3. The gating strategies for flow cytometry analysis of tumors used in this study were shown in Figure S10.

### Western blotting

The cultured cells were washed with PBS and lysed on ice with Cell Lysis Buffer (Yoche, YSD0101) supplemented with Protease and Phosphatase Inhibitor Cocktail (New Cell & Molecular Biotech, P002) for 10 min. Cell lysates were centrifuged at 4℃, 12000 rpm/min for 10 min, and the protein concentration was measured by a BCA kit (share-bio, SB-WB013). Then, cell lysates added by 1×SDS‒PAGE Sample Loading Buffer (Beyotime, P0015) were boiled for 10 min. The protein lysates were separated by 10% or 15% SDS-PAGE and transferred to the NC or PVDF membrane after blocking with 5% non-fat milk. The membrane was incubated overnight with indicated primary antibodies at 4°C and then HRP-conjugated secondary antibodies at RT for 1h. Eventually, the relevant protein was visualized by SuperSignal ECL Chemiluminescence kit (New Cell & Molecular Biotech, P2300) according to the manufacturer's instructions. The results shown were performed in triplicate. Detailed information about antibodies is shown in Table S3. Western blot images were quantitatively analyzed through the Image J software and the quantitative data were added in Figure S11-S12.

### Immunohistochemistry (IHC)

Normal pancreas and pancreatic cancer tissues of humans and mice were fixed with 4% PFA, embedded in paraffin, and sectioned into 5μm-thick slices. Tissue samples were deparaffinized, rehydrated, and antigen retrieved using the standard IHC protocol. After treating with 0.3% hydrogen peroxide for 30 min, tissue samples were blocked for 1 h at room temperature (RT) in 10% BSA. Next, slides were incubated overnight in a humidified chamber at 4°C with primary antibodies listed in Table S3. Then, the sections were incubated with HRP-labeled mouse or rabbit secondary antibody at RT for 1 h. The sections were incubated with DAB substrate solution (Thermo Scientifc, No. S21024- 2) and counterstained in haematoxylin. All sections were observed and photographed using a Carl Zeiss microscope.

The Indica Lab-Area Quantification v2.1.3 module in the Halo v3.0.311.314 analysis software was used to quantify each immunohistochemical image target-area separately. H-Score was calculated (H-SCORE=∑ (PI×I) = (percentage of cells of weak intensity ×1)+(percentage of cells of moderate intensity ×2)+percentage of cells of strong intensity ×3) 33. PI represents the proportion of positive signal area; I stand for color intensity. Then, the Low and High OASL expression was divided by H-score (Low-OASL expression: H-score<5.5; High-OASL expression: H-score>5.5).

### Immunofluorescence

For plasma membrane colocalization experiments, human PDAC cell lines were seeded on coverslips (Ibidi ,80826) coated with poly-lysine overnight. The cells were washed twice with PBS and fixed with 4% paraformaldehyde at RT for 15 min. After washing with PBS for three times, the cell permeability was performed with 0.1% Triton X-100 for 10 min and washed by PBS for three times. The cells were blocked with 1% BSA at RT for 1 h and then were incubated with primary antibodies at 4°C overnight. After washing with PBS for three times, the cells were incubated with fluorescent secondary antibody at RT for 1 h. After washing with PBS for three times, anti-fluorescence quencher containing DAPI was added. Fluorescent images were obtained with confocal microscopy (Carl Zeiss). Detailed information on antibodies is listed in Table S3.

### Co-immunoprecipitation and Mass Spectrometry analysis

Cells expressing or not expressing Flag-*OASL* and HA-*HLA-A* were lysed. Subsequently, cell lysates were incubated with Anti-Flag (Selleck, B26102) or Anti-Ha magnetic beads (Thermo Fisher Scientific, 88836) at 4°C overnight. The complex was washed three times with PBS containing 0.1% Tween and then added by 1×SDS‒PAGE Sample Loading Buffer (Beyotime, P0015). The complex was boiled for 10 min. The co-precipitates were separated using 10% SDS-PAGE and incubated with specific antibodies. The co-precipitates were applied for mass spectrometry analysis (Bioprofile, Shanghai, China). The Mass spectrometry results of proteins interacting with OASL were showed in Table S4.

### Statistical analysis

Data obtained from at least three independent experiments were presented as the mean ± standard deviation (SD). Statistical analysis was performed using GraphPad Prism software 9.0 (GraphPad Software, La Jolla, CA, USA). Student's t-test was used to compare the differences between the two groups. Survival analysis was estimated using Kaplan-Meier methods. Log-rank test was used to calculate statistical differences. P < 0.05 was considered statistically significant.

## Discussion

Pancreatic cancer, especially PDAC, is recognized as one of the most lethal malignancies with strong invasiveness and unsatisfactory therapeutic effects 46. The strategy of immune checkpoint blockade (ICB) fails to provide remarkable clinical benefit for the majority of patients with PDAC 47. The terrible situation is primarily attributed to the disrupted immune system caused by cunning pancreatic cancer cells. Therefore, it is urgent for us to identify and eliminate the accomplices who assist the immune escape of tumor cells. We identified OASL as the key molecule associated with low infiltration of CTLs by MCP-counter algorithms and WGCNA analysis. Currently, The MCP-counter algorithms are widely applied for calculating the abundance of immune cells in the tumor microenvironment (TME) 31. The genes highly co-expressed in cancer can be identified through WGCNA analysis 36. We found that the expression of OASL was remarkably increased in PDAC cells compared with normal pancreatic cells, and OASL was correlated to the unfavorable prognosis of PDAC patients. OASL is a member of the antiviral protein OAS family, which plays crucial roles in the innate and adaptive immune responses to viruses 48. However, the anti-tumor immunity of OASL has rarely been reported. Our study demonstrated that *OASL*-knockdown increased MHC-I levels by inhibiting NBR1-mediated autophagy-lysosomal degradation in PDAC, contributing to the infiltration of CD8^+^T cells and tumor suppression.

MHC-I plays an important role in antigen presentation. The MHC-I complexes which are loaded with nascent tumor antigen peptides are delivered to the surface of tumor cells for the recognition of CD8^+^T cells 49. However, the loss of MHC-I is caused by sneaky tumor cells to circumvent surveillance from the immune system. The decreased CD8^+^T cell infiltration is closely associated with the loss of MHC-I in the progression of cancer, but MHC-I gene mutations are rare in pancreatic cancer, suggesting that the downregulation of MHC-I may be the principle underlying mechanism for immune escape in PDAC. A previous study revealed that SUSD6, TMEM127 and MHC-I formed a ternary complex that recruited WWP2 for MHC-I ubiquitination and lysosomal degradation, shrinking the amount of CD8^+^T cells. A membrane-associated MHC-I inhibitory axis provides a potential therapeutic target for "cold tumor", including leukemia and pancreatic cancer 50. Another study demonstrated that knockdown or drug inhibition of the glucocorticoid receptor (GR) facilitated the increasing expression of MHC-I, thereby enhancing the infiltration of CTLs and the sensitivity of pancreatic cancer to immune checkpoint blockade 51. In our study, we demonstrated that the decreased MHC-I levels caused by OASL can be reversed by BafA1. CQ, recognized as an inhibitor of autophagy, elevates lysosomal pH and inhibits the autophagosome-lysosome fusion, preventing the maturation of autophagosome 52. CQ has previously been widely applied to treatment of malaria, but recent studies have shown that CQ is a promising anti-tumor drug. CQ increases macrophage lysosomal pH, stimulating p38 and NF-κB activation, leading to the polarization of tumor-associated macrophages (TAMs) to tumor-killing M1 phenotype. Moreover, CQ-reset macrophages reshape the tumor immune microenvironment by reducing the immunosuppressive infiltration of bone marrow-derived suppressor cells and Treg cells, thereby enhancing anti-tumor T cell immunity 53. Hydroxychloroquine, a derivative of chloroquine, exerts a vigorous effect on therapies with PDAC 54, 55. It has been reported that the combination of CQ and dual ICB (anti-PD-1 and anti-CTLA4 antibodies) significantly reduces tumor weight and increases the infiltration of T cells in PDAC 28. In our study, OASL promotes autolysosome-mediated degradation of MHC-I in PDAC. Therefore, we propose that PDAC patients with high expression of OASL may respond better to a combination therapy of CQ and dual ICB, suggesting a new direction for anti-tumor therapy development.

Autophagy, an essential cellular catabolic mechanism, is mainly responsible for recycling and degrading unnecessary metabolites or dysfunctional cellular components in autophagolysosome 56. The disruption of autophagy leads to impaired homeostasis, which may cause various diseases, including neurodegeneration, metabolic disorders and cancer 57. The previous research indicated that autophagy damaged infiltration of CD8^+^T cells in PDAC 28. OASL has been shown to be involved in antiviral immune response and bacterial autophagy 19. Subsequently, we identified a novel mechanism in which OASL interacts with NBR1, facilitating MHC-I ubiquitination and redirecting MHC-I into autophagy. Zeng et al. found that basic polymerase 1 (PB1) encourages the degradation of ubiquitinated MAVS through NBR1 in autophagosomes, which inhibits the innate immune response to Influenza A virus (IAV) 58. NBR1 binds to the ubiquitination substrate in selective autophagy and promotes its degradation in lysosomes. It was reported that MHC-I was degraded by binding to NBR1 and entering the autophagy process, thus promoting pancreatic cancer immune escape 28.

Our study mainly focused on that OASL facilitates the degradation of MHC-I and slack of anti-tumor adaptive immunity derived from CD8^+^T cells through NBR1-mediated autophagy-lysosomal degradation. However, the specific domain of interaction between OASL and NBR1 remains to be elucidated. Furthermore, we can attempt to take advantage of ICB therapy based on OASL targeted therapy in PDAC in the future. Finally, several mechanisms through which OASL fosters PDAC proliferation and immune evasion await further exploration through additional experiments. PDAC potentially dodges immune cell detection by exploiting immune checkpoint features and releasing cytokines that dampen immune cell function. Investigating how OASL contributes to PDAC immune evasion will be the primary focus of our future research efforts.

## Conclusions

In our study, we identified OASL as a crucial molecular related to immune evasion, controlling the expression of MHC-I. Targeting OASL or NBR1 can partially restore the expression of MHC-I through restraining autophagy-lysosomal degradation, enhancing the ability of the antitumor immune responses and prolonging survival in PDAC. Our data indicate that OASL regulates adaptive immune processes by influencing CTLs. Mechanistically, the function of CD8^+^T cells was abolished by OASL through NBR1-mediated MHC-I degradation in autophagy-lysosomal in PDAC. Therefore, the inhibition of OASL could be a novel immunotherapy strategy for patients with PDAC, and build up a favorable basis for further research to improve the outcome of PDAC patients.

## Supplementary Material

Supplementary figures and tables.

## Abbreviations

PDAC: Pancreatic ductal adenocarcinoma
CTLs: cytotoxic T lymphocytes
OASL: oligoadenylate synthetase-like
ICB: immune checkpoint blockade
MHC-I: major histocompatibility complex class I
ISG: interferon-stimulated gene
ATG4: autophagy-related protein 4
RT: room temperature
MCP-counter: Microenvironment Cell Populations-counter
WGCNA: Weighted Gene Co-expression Network Analysis
IHC: immunohistochemical
BafA1: bafilomycin
NBR1: neighbor of BRCA1 gene 1
TAMs: tumor-associated macrophages
