# Supplementary material for: OASL promotes immune evasion in pancreatic ductal adenocarcinoma by enhancing autolysosome-mediated degradation of MHC-I
Source: Theranostics. 2025 Jan 13;15(6):2104–20. doi: 10.7150/thno.103494 (PMC11840728; doi:10.7150/thno.103494)
Supplement: Supplementary file 1 — Supplementary figures and tables. [file thnov15p2104s1.pdf]

Supplementary Figure and figure legends

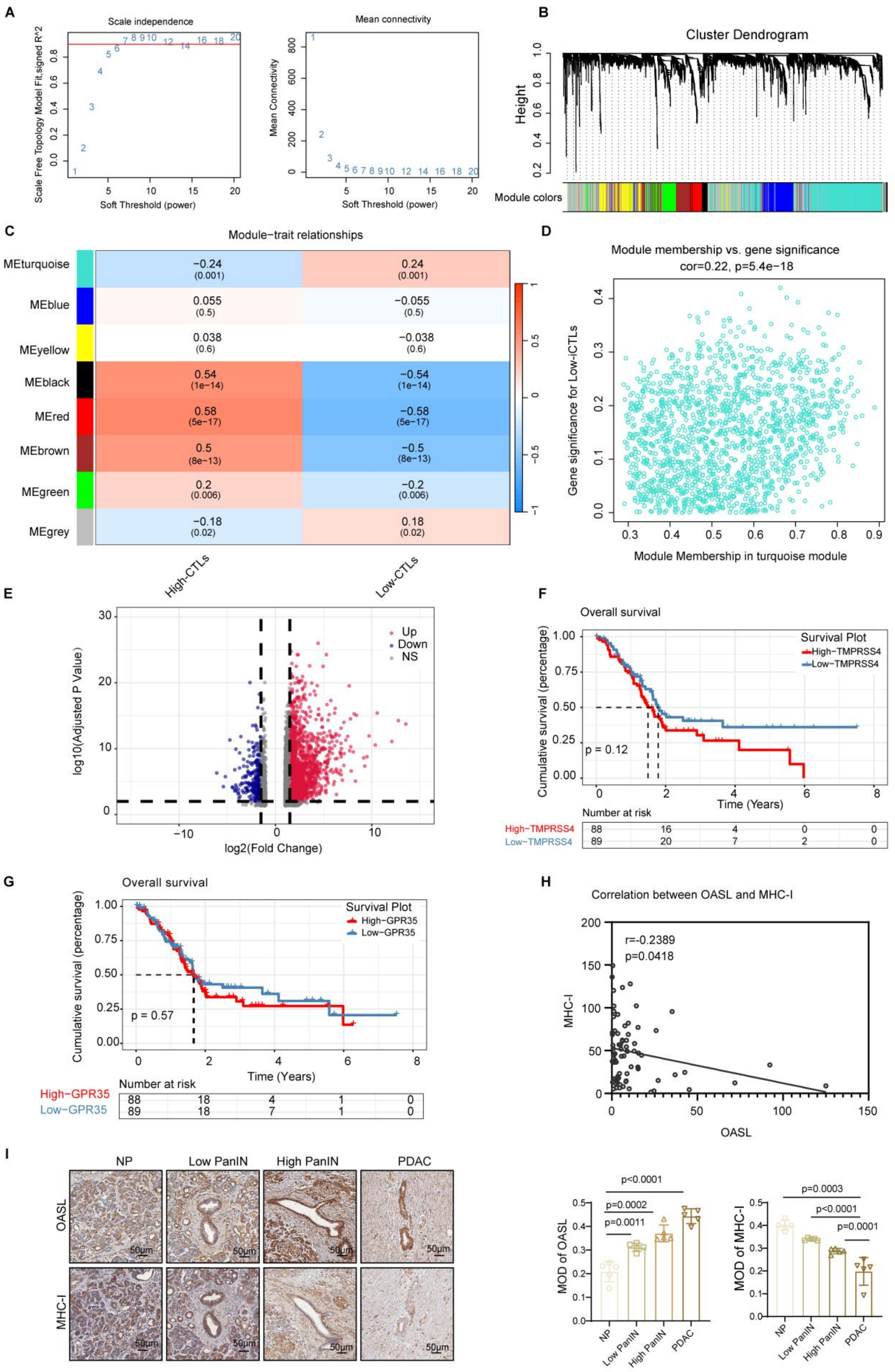

Figure S1. Identification of key molecules disturbing immune evasion. (A) Scale

independence (left) and mean connectivity (right) were utilized to screen the optimal soft threshold of WGCNA (power = 6). (B) Gene clustering dendrograms. (C) Heat map of correlations between gene modules and clinical features (High-CTL, Low-CTL). The correlation and p-value were marked in each grid. (D) Correlation analysis of Low-CTL and turquoise module in PAAD. cor = 0.22, p = 5.4e-18. (E) Volcano plots of differentially expressed genes between pancreatic tumor and normal samples using TCGA-GTEx. The red points represent up-regulated, and green points represent down-regulated genes. The differences are set as $|\log_2$ (Fold Change)$|>1.5$, p-adj<0.01, baseMean > 100. (F) Kaplan-Meier curves of OS differences layered by the High-*TMPRSS4* and Low-*TMPRSS4* groups from TCGA data set (n = 88, n = 89). (G) Kaplan-Meier curves of OS differences layered by the High-*GPR35* and Low-*GPR35* groups from TCGA data set (n = 88, n = 89). The p-value was calculated using Log Rank test. (H) Correlation analysis of OASL and MHC-I expression in a tissue microarray from Renji cohort (n=73) (p-value = 0.0418, cor = -0.2389). (I) The immunohistochemical images for OASL and MHC-I in normal pancreas (NP), Low pancreatic intraepithelial neoplasia (PanIN), High PanIN and PDAC in humans. (Scale bar, 50 μm) (left). The mean optical density (MOD) was used for evaluating the protein expression of OASL and MHC-I by image J (right)(n = 5). Bars represent mean ± standard deviation. The p-value was calculated using an unpaired T-test in Figure S1I.

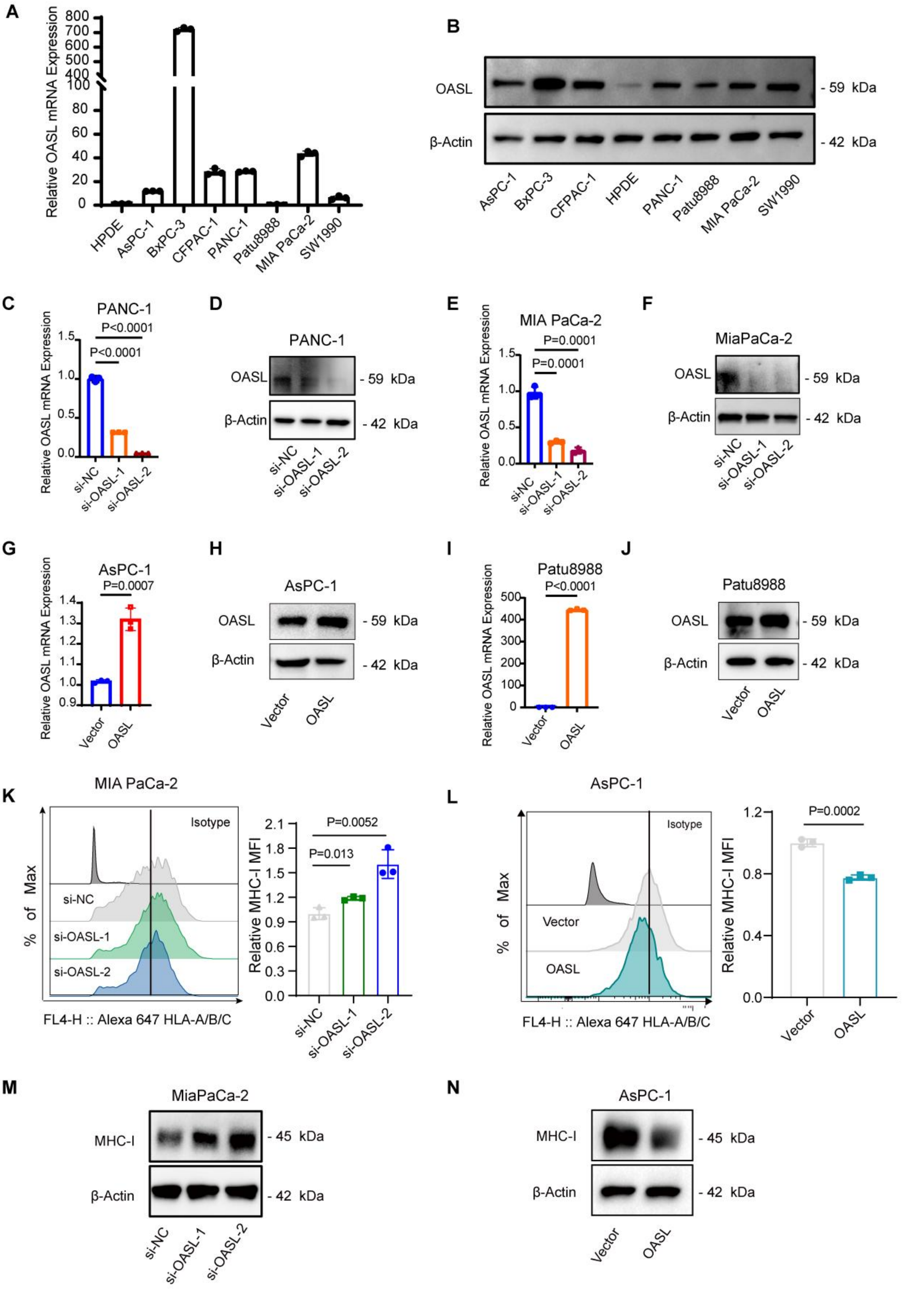

Figure S2. OASL represses the expression of MHC-I in PDAC cell lines. (A-B) The expression of *OASL* in human PDAC cell lines was detected by qPCR (A) and Western blot analysis (B).n = 3 biological replicates. Representative images were shown. (C-F) The efficiency of *OASL*-knockdown was validated by qPCR and Western blot analysis in PANC-1 cells (C-D) and

MIA PaCa-2 cells (E-F). n = 3 biological replicates. Representative images were shown. (G-J) qPCR and Western blot detection of *OASL*-overexpression in AsPC-1 cells (G-H) and Patu8988 cells (I-J). n = 3 biological replicates. Representative images were shown. (K-L) The expression of HLA-A/B/C on the surface of PDAC cell lines was measured by flow cytometry in MIA PaCa-2 cells with *OASL*-knockdown (K) and AsPC-1 cells with *OASL*-overexpression (L) (n = 3, per group). (M-N) Western blot analysis of MHC-I in MIA PaCa-2 cells with *OASL*-knockdown (M) and AsPC-1 cells with overexpression of *OASL* (N). n = 3 biological replicates. Representative images were shown. Bars represent mean ± standard deviation. The p-value was calculated using an unpaired T-test in Figure S2C, S2E, S2G, S2I, S2K-S2L.

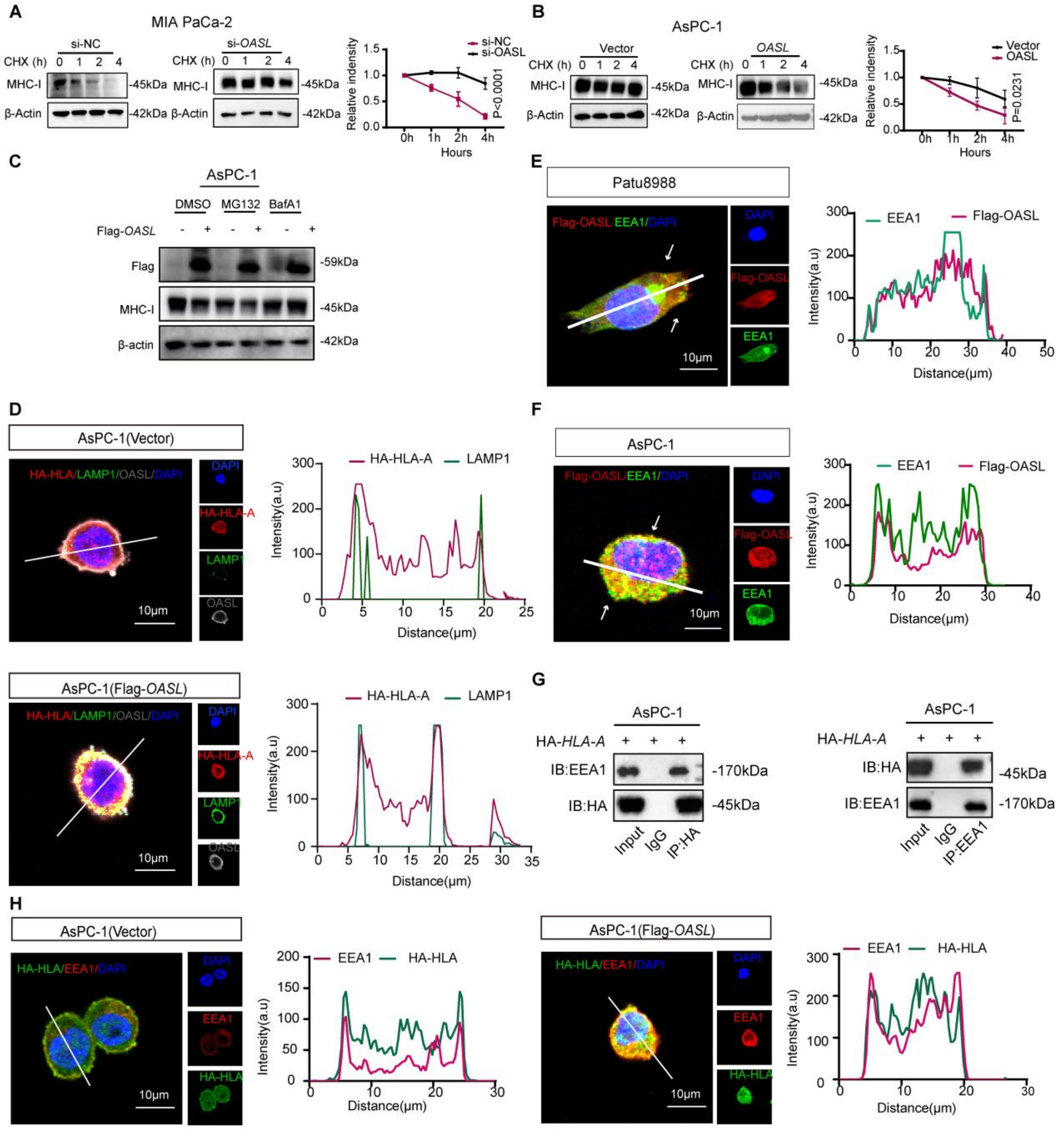

Figure S3. Loss of MHC-I is caused by OASL in the early endosome. (A-B) The half-life of MHC-I was evaluated in MIA PaCa-2 cells with *OASL*-knockdown (A) and AsPC-1 cells with overexpression of *OASL* (B) treated with CHX (20μM) at the specific time point by Western blot (left).The protein half-life curves were obtained by quantifying relative intensities (right). n = 3 biological replicates. Representative images were shown. (C) AsPC-1 cells with Flag-*OASL* were treated with dimethyl sulfoxide (DMSO), proteasome inhibitor MG132 (20μM), lysosomal inhibitor BafA1 (20μM) for 4 h and the expression of MHC-I was evaluated by Western blot. n = 3 biological replicates. Representative images were shown. (D) AsPC-1 cells stably expressing HA-*HLA-A* was transfected with Flag-*OASL* and Vector and were stained for HA-HLA-A (red), LAMP1 (green) and OASL (gray). Cell nucleus (blue) was stained with DAPI. The colocalization

was visualized by confocal microscopy. Representative immunofluorescence images were shown (Scale bar, 10 μm)(left). Image analysis of immunofluorescenct staining intensity across the line was shown in the right panel. (E-F) Patu8988 cells (E) and AsPC-1 cells (F) was transfected with Flag-*OASL* and were stained for Flag-OASL (red), EEA1 (green). Cell nucleus (blue) was stained with DAPI. The colocalization of Flag-OASL and EEA1 was visualized by confocal microscopy. Representative immunofluorescence images were shown (Scale bar, 10 μm) (left). Image analysis of immunofluorescenct staining intensity across the line were shown in the right panel. (G) AsPC-1 cells were stably transfected with HA-HLA. The whole-cells were immunoprecipitated with anti-HA beads and followed by Western blot with antibodies against the EEA1 (left).The whole-cells were immunoprecipitated with anti-EEA1 beads and followed by Western blot with antibodies against the HA (right). n = 3 biological replicates. Representative images were shown. (H) AsPC-1 cells stably expressing HA-*HLA-A* was transfected with Flag-*OASL* and Vector and were stained for EEA1 (red) and HA-HLA (green). The cell nucleus (blue) was stained with DAPI. The colocalization of EEA1 and HA-HLA-A was visualized by confocal microscopy. Representative immunofluorescence images were shown (Scale bar, 10 μm) (left). Image analysis of immunofluorescenct staining intensity across the line was shown in the right panel. Bars represent mean ± standard deviation. The p-value was calculated using Two-way ANOVA in Figure S3A-B.

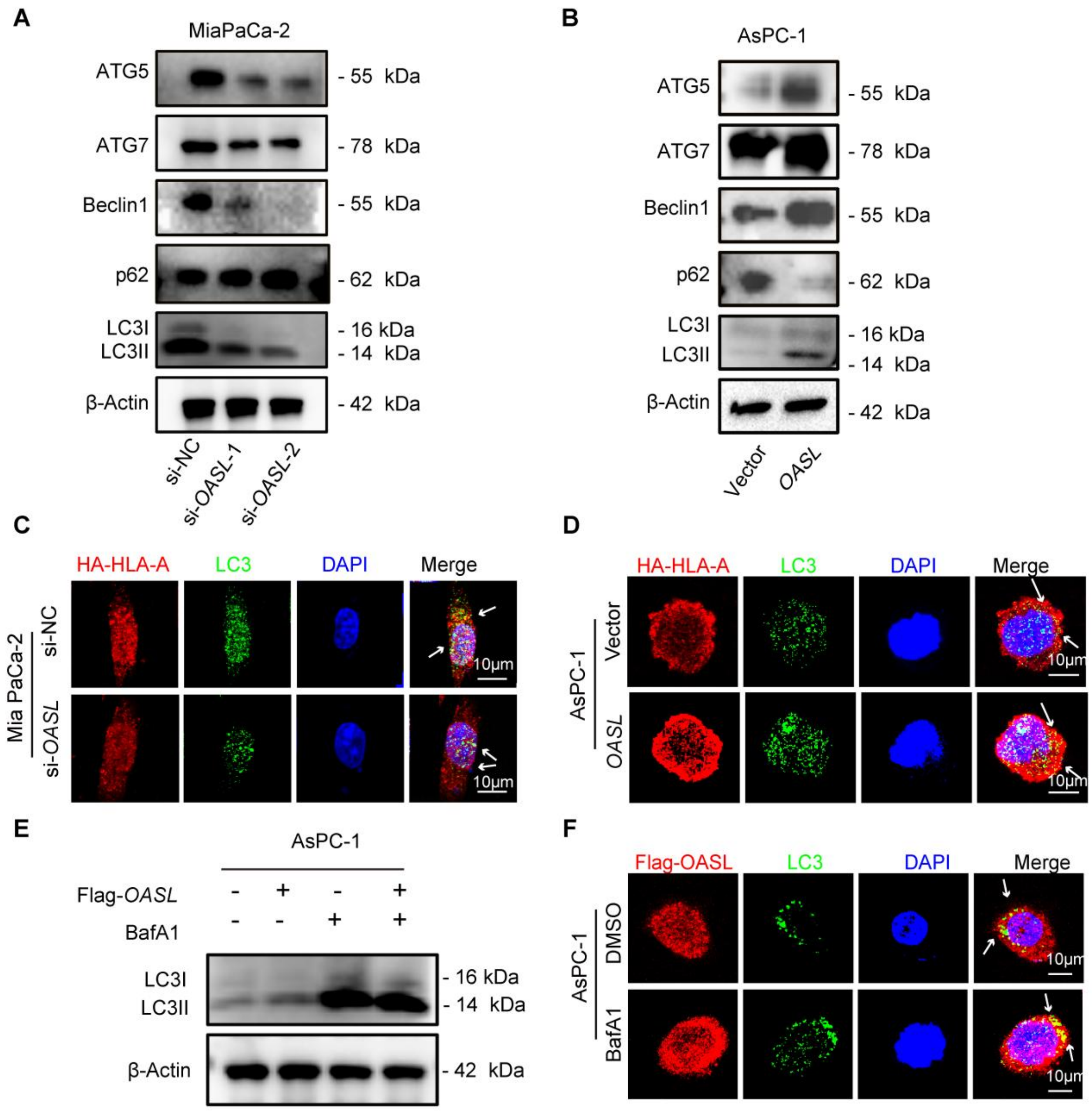

Figure S4. OASL participates in the autophagy. (A-B) The expression levels of ATG5, ATG7, Beclin1, p62 and LC3 (LC3-I and -II) were detected by Western blot analysis in MIA PaCa-2 cells with *OASL*-knockdown (A) and *OASL*-overexpression AsPC-1 cells (B). n = 3 biological replicates. Representative images were shown. (C-D) MIA PaCa-2 cells with *OASL*-knockdown (C) and AsPC-1 cells with Flag-*OASL* (D) were stably transfected by HA-*HLA-A* and stained for HLA-A-HA (red) and LC3 (green). The cell nucleus (blue) was stained with DAPI. The colocalization was visualized by confocal microscopy. Representative immunofluorescence images were shown. (Scale bar, 10 μm). (E) Western blot analysis of LC3 (LC3-I and -II) level in AsPC-1 cells with Flag-*OASL* upon treatment with or without BafA1. n = 3 biological replicates. Representative images were shown. (F) AsPC-1 cells stably expressing HA-*HLA-A* were transfected with Flag-*OASL* and were treated with DMSO (4 h) or BafA1 (4 h) were stained for

Flag-OASL (red) and LC3 (green). The cell nucleus (blue) was stained with DAPI. The colocalization was visualized by confocal microscopy. Representative immunofluorescence images were shown (Scale bar, 10 μm).

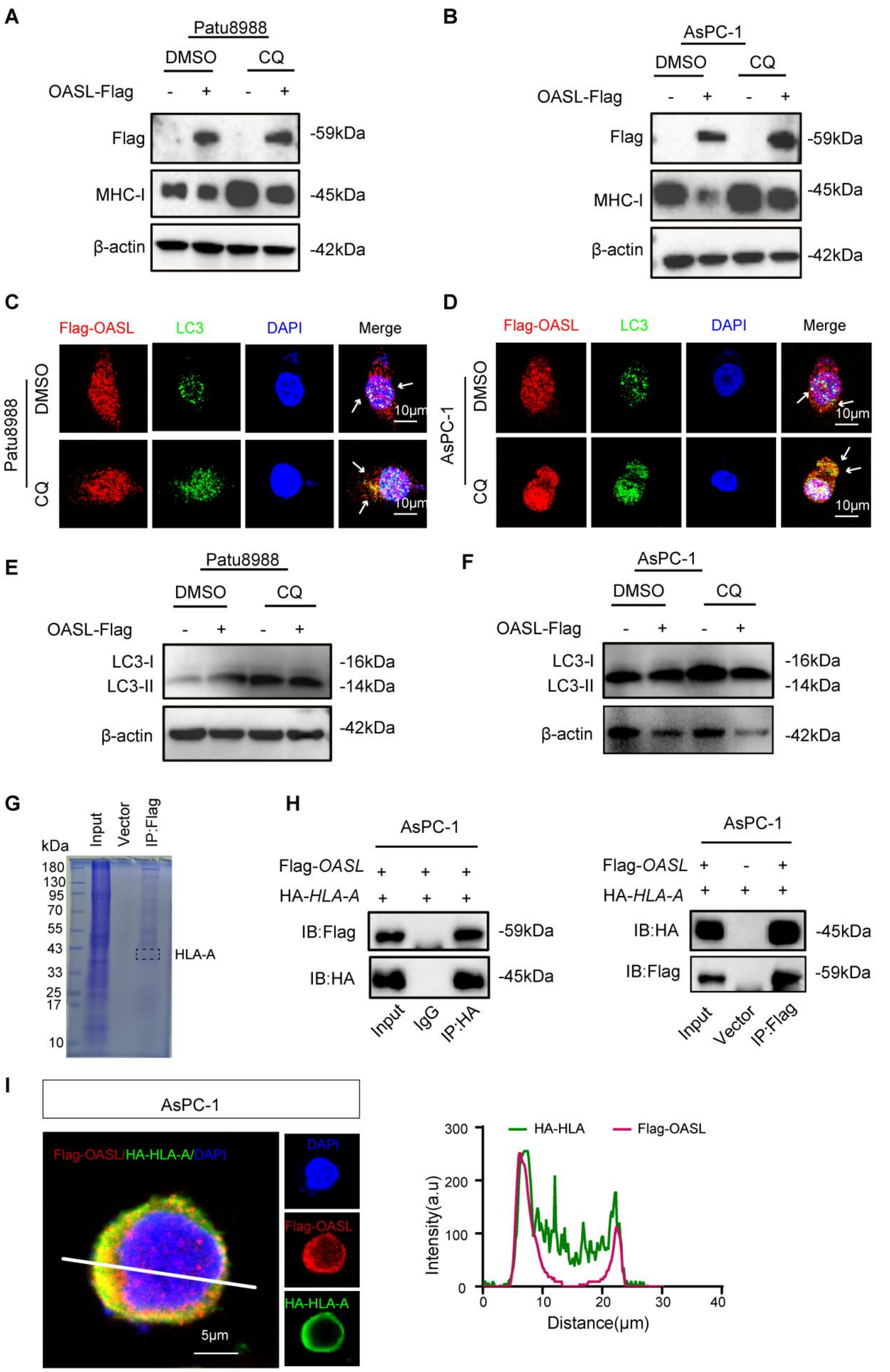

Figure S5. OASL promotes the degradation of MHC-I by autolysosome. (A-B) Patu8988 cells (A) and AsPC-1 cells (B) with overexpression of *OASL* were treated with dimethyl sulfoxide

(DMSO) and an inhibitor of autophagy CQ (20μM) for 4 h, and the expression of MHC-I was evaluated by Western blot. n = 3 biological replicates. Representative images were shown. (C-D) Patu8988 cells (C) and AsPC-1 cells (D) with Flag-*OASL* were treated with DMSO (4 h) or CQ (4 h) and stained for Flag-OASL (red) and LC3 (green). The cell nucleus (blue) was stained with DAPI. The colocalization was visualized by confocal microscopy. Representative immunofluorescence images were shown (Scale bar, 10 μm). (E-F) Western blot analysis of LC3 (LC3-I and -II) levels in Patu8988 cells (E) and AsPC-1 cells (F) expressing Flag-*OASL* upon treatment with or without CQ. n = 3 biological replicates. Representative images were shown. (G) Immunopurification and mass spectrometry of OASL-containing protein complexes. Cellular extracts from Patu8988 cells expressing Flag-*OASL* were immunopurified with anti-Flag affinity beads and eluted with Flag peptide. The elutes were resolved on SDS-PAGE and stained with Coomassie Brilliant Blue. The protein bands on the gel were analyzed by mass spectrometry. (H) The relationship between OASL and HLA-A was analyzed by Co-IP analysis. Flag-*OASL* was found in AsPC-1 cells transfected with HA-HLA. The whole-cells were immunoprecipitated with anti-HA beads and followed by Western blot with antibodies against the Flag. Vector and Flag-*OASL* were transfected in AsPC-1 cells with HA-HLA. The whole-cells were immunoprecipitated with anti-Flag beads and followed by Western blot with antibodies against the HA. n = 3 biological replicates. Representative images were shown. (I) AsPC-1 cells stably expressing HA-*HLA-A* was transfected with Flag-*OASL* and stained for HA-HLA-A (green) and Flag-OASL (red). The cell nucleus (blue) was stained with DAPI. The colocalization was visualized by confocal microscopy. Representative immunofluorescence images were shown (Scale bar, 5 μm)(left). Image analysis of immunofluorescenct staining intensity across the line was shown in the right panel.

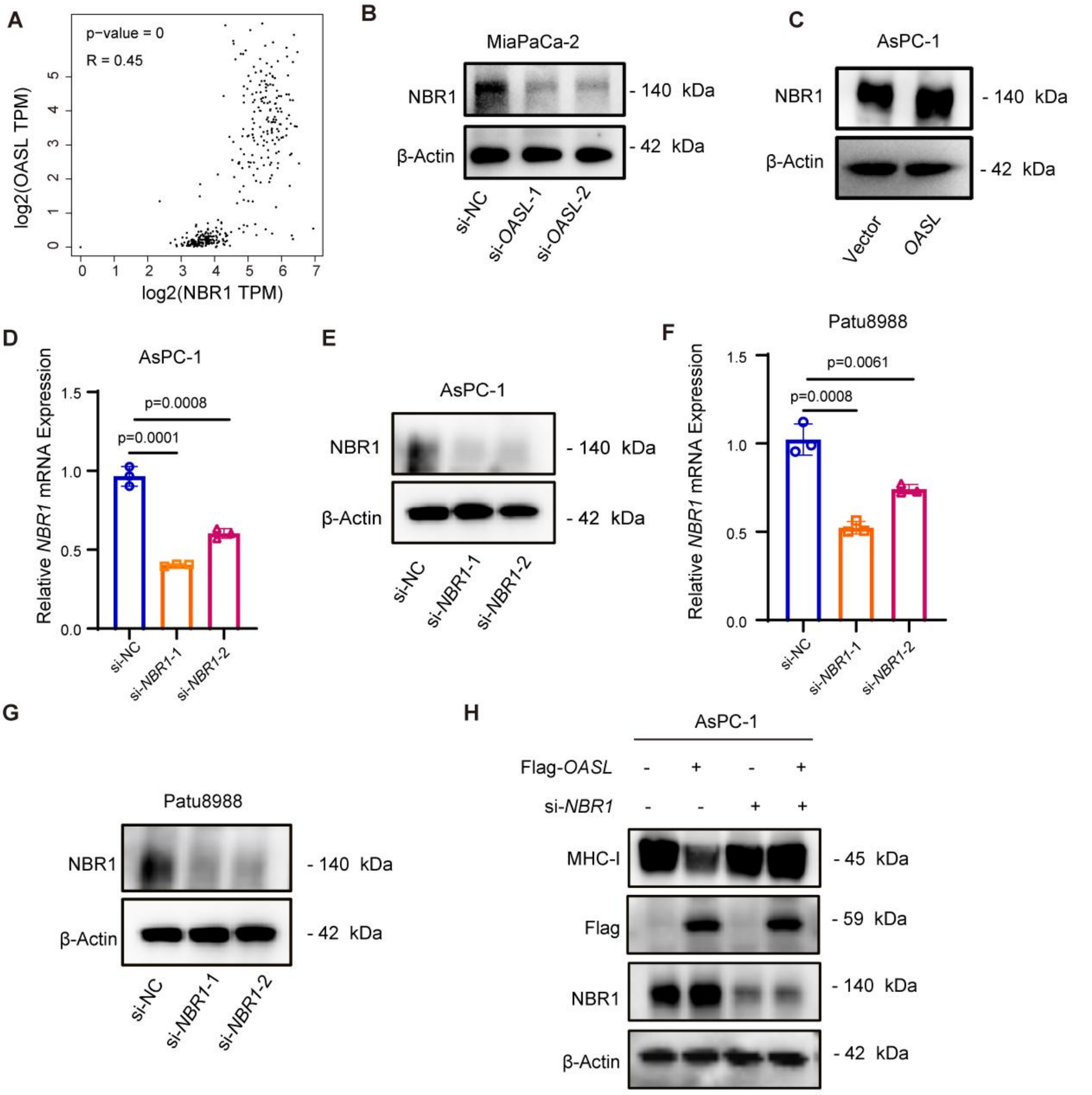

Figure S6. The expression of NBR1 and efficiency of knockdown-*NBR1* in PDAC cell lines. (A) Correlation analysis of *NBR1* and *OASL* expression in pancreatic cancer from GEPIA2 databases (p-value = 0, cor = 0.45). (B-C) The expression of NBR1 was measured by Western blot in MIA PaCa-2 cells with *OASL*-knockdown (B) and AsPC-1 cells with Flag-*OASL* (C).n = 3 biological replicates. Representative images were shown. (D-G) The efficiency of knockdown-*NBR1* was validated by qPCR and Western blot analysis in AsPC-1 cells and Patu8988 cells (n = 3, per group). n = 3 biological replicates. Representative images were shown. (H) Western blot analysis of MHC-I expression levels in AsPC-1 cells with Flag-*OASL* with or without *NBR1*-knockdown. n = 3 biological replicates. Representative images were shown. Bars represent mean ± standard deviation. The p-value was calculated using an unpaired T-test in Figure S6D and S6F.

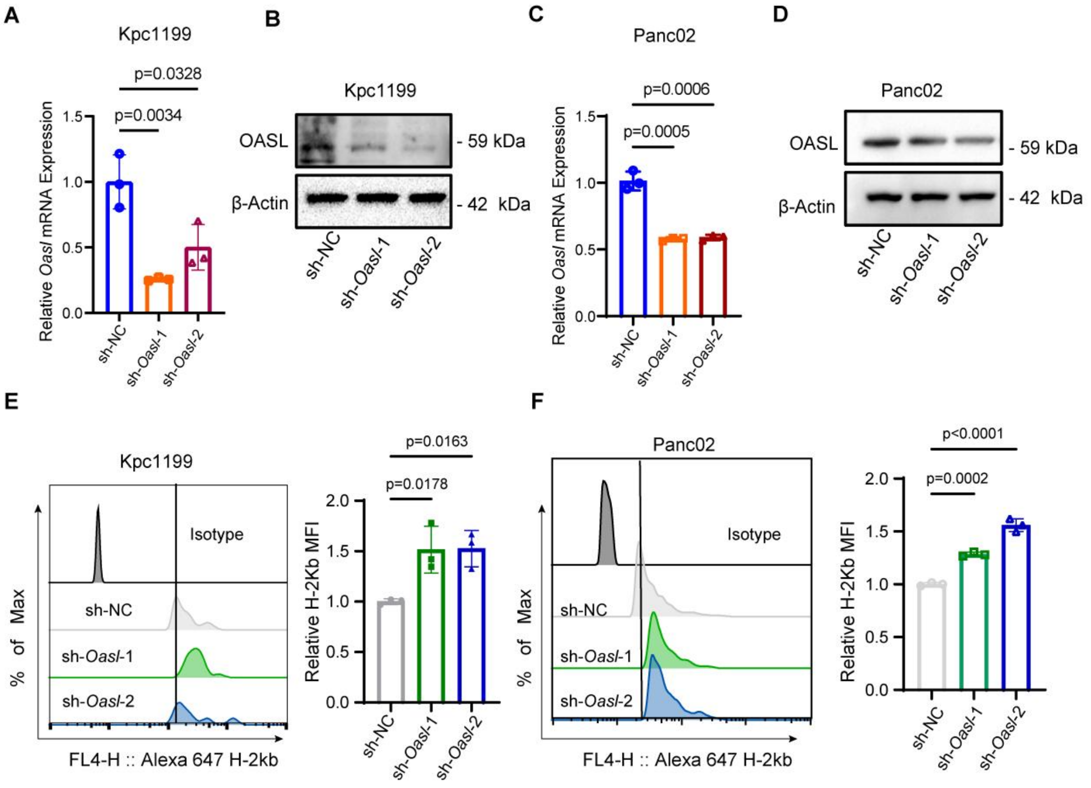

Figure S7. Knockdown of *Oasl* increases MHC-I level in PDAC models. (A-D) The efficiency of *Oasl*-knockdown was validated by qPCR and Western blot analysis in Kpc1199 cells and Panc02 cells (n = 3, per group).n = 3 biological replicates. Representative images were shown. (E-F) The expression of H-2K^{b} on the surface was measured by flow cytometry in Kpc1199 and Panc02 cells treated with knockdown-*Oasl* (n = 3, per group). Bars represent mean ± standard deviation. The p-value was calculated using an unpaired T-test in Figure S7E-F.

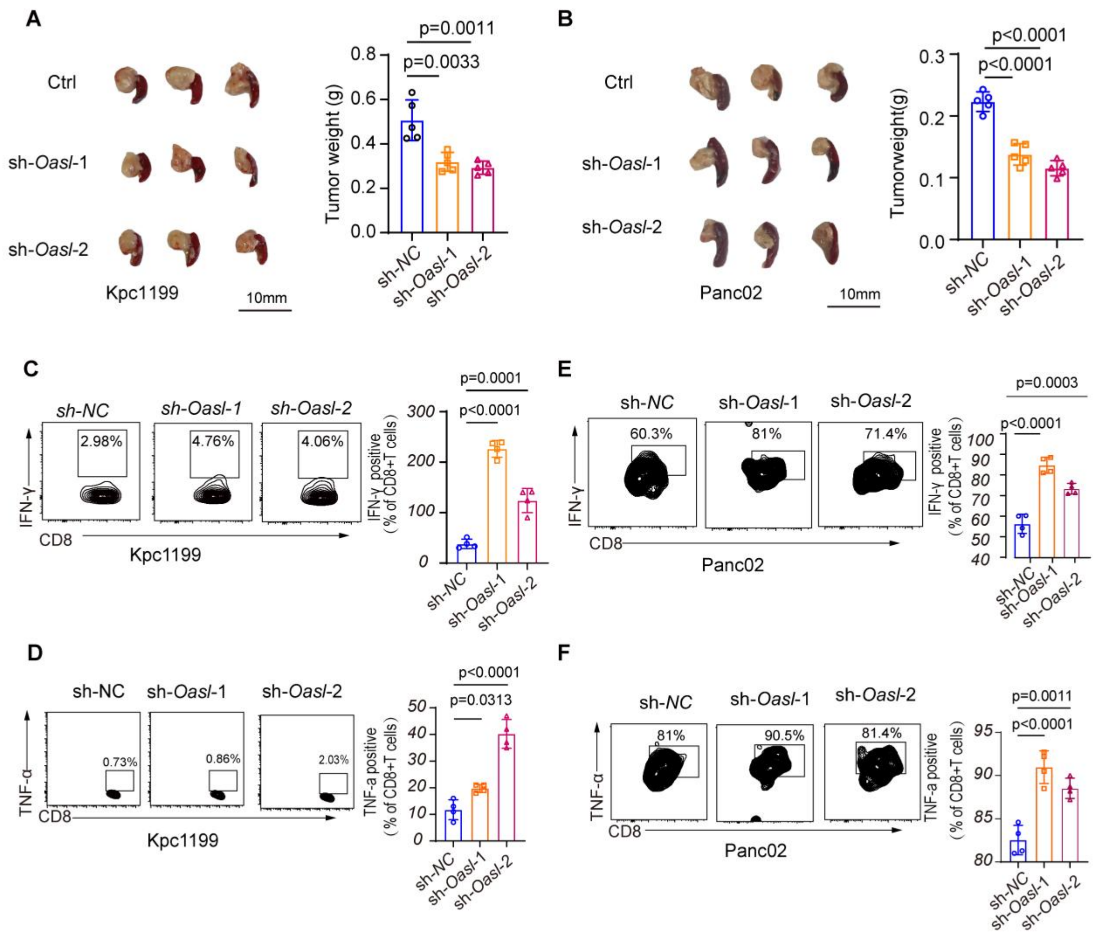

Figure S8. OASL inhibits anti-tumor immune response by decreasing the proliferation of PDAC and activation of CD8⁺T cell *in vivo*. (A-B) Representative images and weight of pancreatic tumor in situ in Kpc1199 cells (A) and Panc02 cells (B) bearing sh-NC, sh-*Oasl*-1 and sh-*Oasl*-2. The orthotopic pancreatic tumors were collected on day 23 (n = 5, per group). (C-D) The proportion of IFN-γ (C) and TNF-α (D) tumor-infiltrating CD8⁺T cells was analyzed by flow cytometry in the immune microenvironment of mice that were orthotopically injected with Kpc1199 cells bearing sh-NC, sh-*Oasl*-1 and sh-*Oasl*-2 (n = 4, per group). (E-F) The proportion of IFN-γ (E) and TNF-α (F) tumor-infiltrating CD8⁺T cells was analyzed by flow cytometry in the immune microenvironment of mice that were orthotopically injected with Panc02 cells bearing sh-NC, sh-*Oasl*-1 and sh-*Oasl*-2 (n=4, per group). Bars represent mean ± standard deviation. The p-value was calculated using an unpaired T-test in Figure S8A-F.

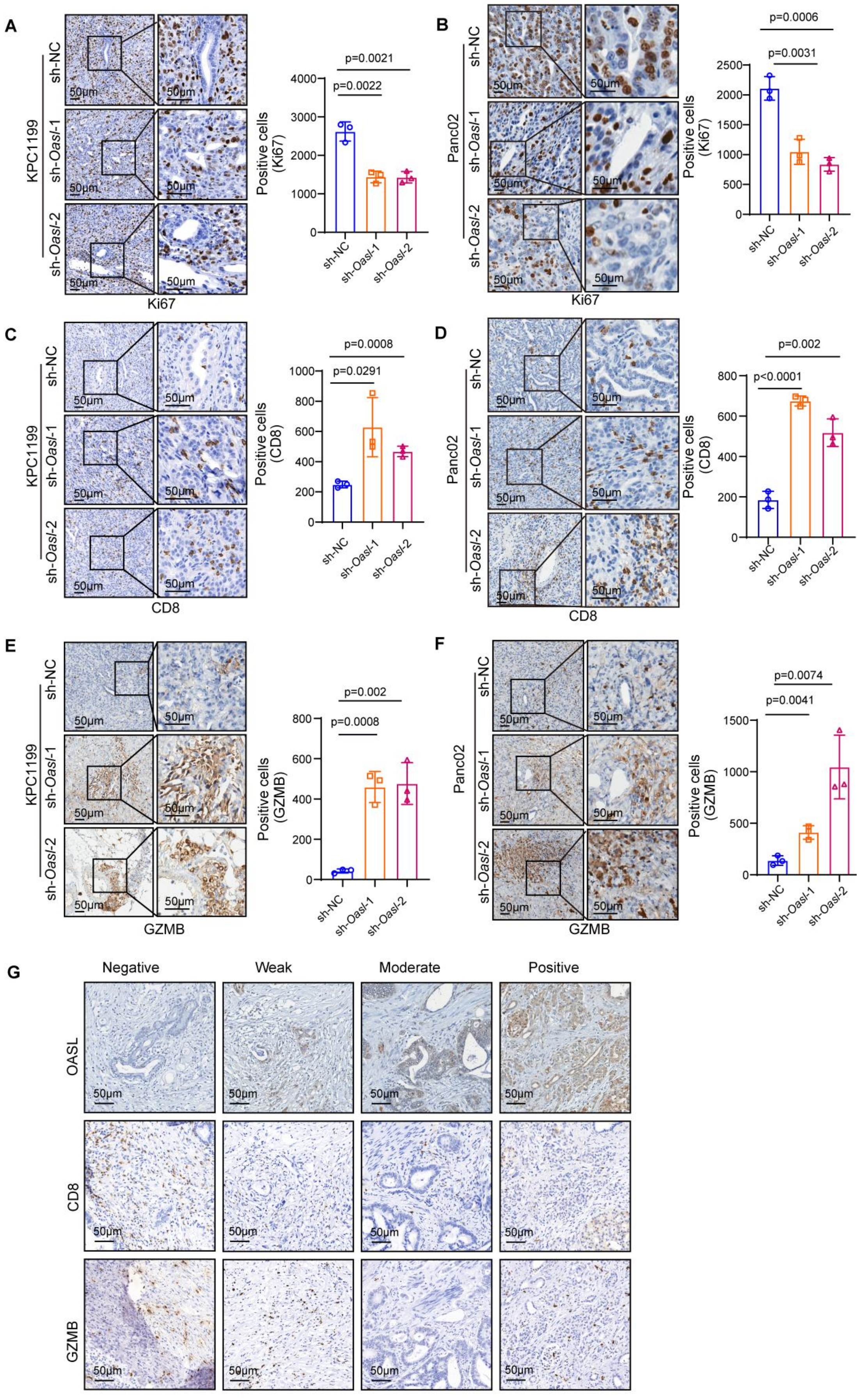

A
KPC1199
sh-NC
sh-Oasl-1
sh-Oasl-2
50μm
Ki67
p=0.0021
p=0.0022
Positive cells (Ki67)
B
Panc02
p=0.0006
p=0.0031
C
CD8
p=0.0008
p=0.0291
Positive cells (CD8)
D
p=0.002
p<0.0001
E
GZMB
p=0.002
p=0.0008
Positive cells (GZMB)
F
p=0.0074
p=0.0041
G
Negative
Weak
Moderate
Positive
OASL
CD8
GZMB

Figure S9. ***OASL*****-knockdown inhibited progression of PDAC and facilitated the filtration of CD8 and GZMB.** The immunohistochemical staining of Ki67 (A-B), CD8 (C-D) and GZMB (E-F) from pancreatic tumors with sh-NC, sh-*Oasl*-1, sh-*Oasl*-2 groups in Kpc1199 and Panc02 cells was shown. (G) IHC staining analysis for OASL, CD8 and GZMB in a tissue microarray (Scale bar, 50 μm). Bars represent mean ± standard deviation. The p-value was calculated using an unpaired T-test in Figure S9A-F.

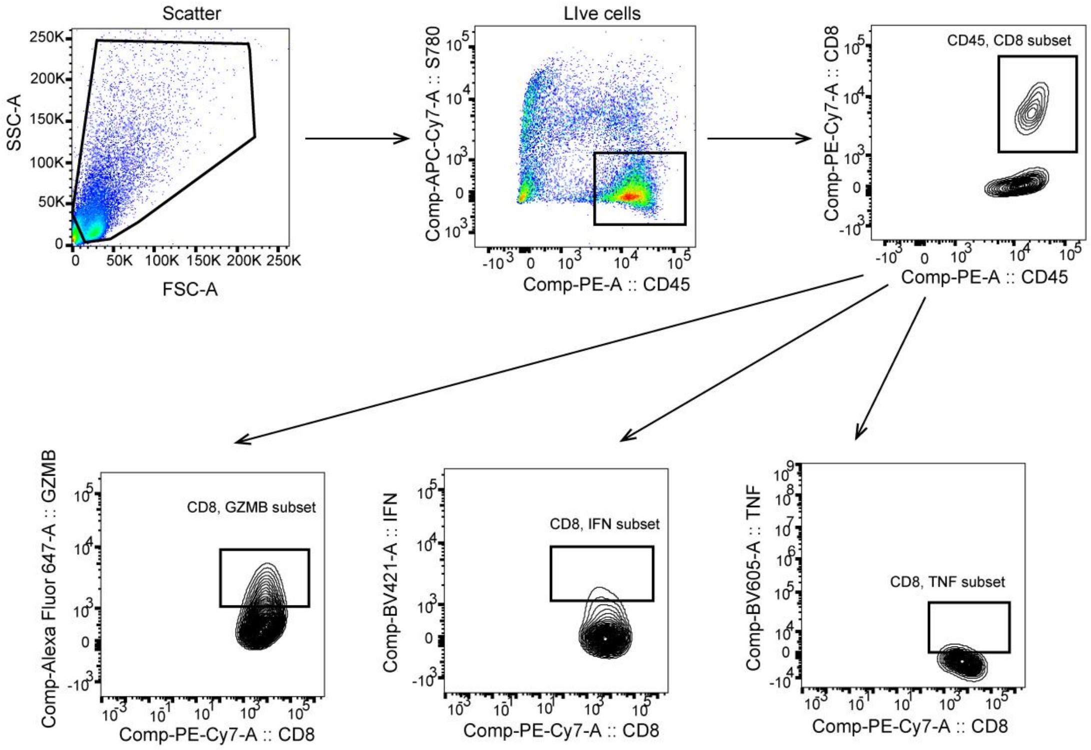

Figure S10. The gating strategies for flow cytometry analysis of tumors used in this study.

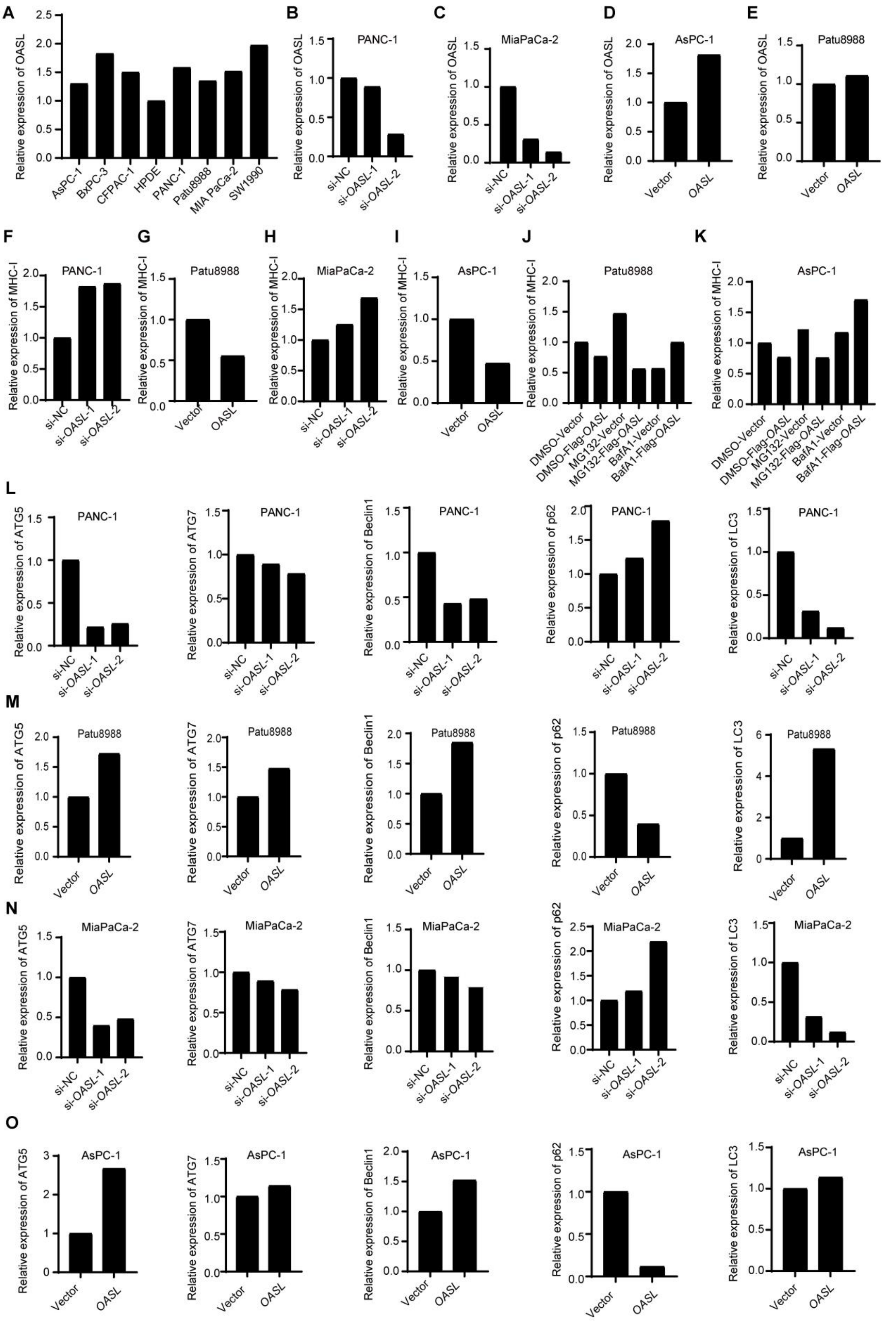

Figure S11. Western blot images were quantitatively analyzed. (A) The densitometry analysis data of protein level of OASL of Figure S2B. (B-E) The densitometry analysis data of protein

level of OASL of Figure S2D, S2F, S2H and S2J. (F-I) The densitometry analysis data of protein level of MHC-I of Figure 2F, 2G, S2M and S2N. (J-K) The densitometry analysis data of protein level of MHC-I of Figure 3C and Figure S3C. (L-O) The densitometry analysis data of protein levels of ATG5, ATG7, Beclin1, p62 and LC3 of Figure 4A-B and Figure S4A-B.

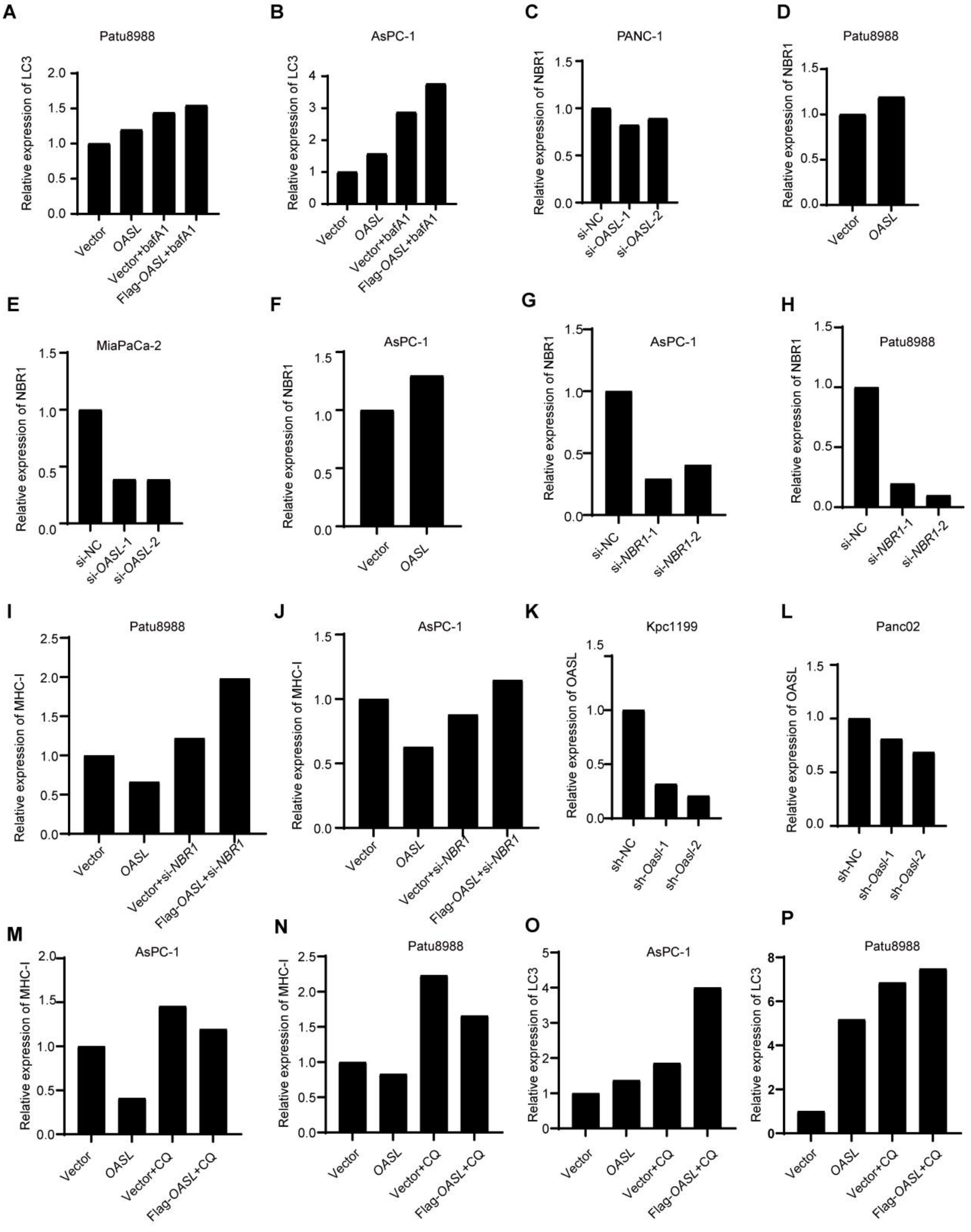

Figure S12.Western blot images were quantitatively analyzed. (A-B) The densitometry analysis data of protein level of LC3 of Figure 4E and Figure S4E. (C-F) The densitometry analysis data of protein level of NBR1 of Figure 5F-G, Figure S6B-6C. (G-H) The densitometry analysis data of protein level of NBR1 of Figure S6E and S6G. (I-J) The densitometry analysis data of protein level of MHC-I of Figure 5H and Figure S6H. (K-L) The densitometry analysis data of protein level of OASL of Figure S7B and Figure S7D. (M-N) The densitometry analysis

data of protein level of MHC-I of Figure S5A-B. (O-P) The densitometry analysis data of protein level of LC3 of Figure S5E-F.

Table S1 Proteins that interact with HLA-A from BioGRID database

Official Symbol Interactor with HLA-A						
HLA-A	SDHA	EBAG9	RAC3	HERC2	RHOA	HLA-B
DERL1	K1	ELOVL5	RHOB	SCLT1	RHOJ	HLA-C
LILRB1	EGLN3	ERGIC1	RHOD	TMEM17	RHOQ	HLA-E
MAGEA4	MAPK6	EZR	RHOU	ATXN3	nsp6	ATP1B4
MAGEA1	MEX3C	GJA1	RHOH	PCDHB15	CBL	PTCH1
KIR3DL2	ADSS	GJD3	RND1	Arhgef17	EZH2	ZACN
TRA	BMPR1A	HSD17B11	RND2	Cep85l	S	IFI16
TRB	CDK9	HSD3B7	RND3	PTPN5	PRNP	E
TAPBP	TGFB1	LAMP2	RAC2	HLA-G	Rab18	ORF3b
UBC	RNF4	LAMP3	RHOC	ADAM33	ORF7b	M
TAP1	GPC1	LAMTOR1	RHOF	TCTN2	ORF10	Nsp4
TRAM1	HRAS	LYN	RHOV	CARKD	CLEC16A	HSCB
SEC61A1	NRAS	MARCKS	CANX	SLC39A5	SPOP	CCR9
BCAP31	BET1	METTL7A	FOLR1	TMPRSS3	ESYT1	EP300
LMAN1	LAMP1	OCLN	MYCN	PTPRO	DSCAM	TMPRSS1B
TAP2	KIAA1429	PANX1	MYC	DLST	RNF149	NRP1
Env	TMEM41B	RAB11A	VCP	Cebpb	RPA1	TMPRSS2
MMS19	KRAS	RAB2A	ORF6	UBE2H	RPA2	CTSL
CALR	CYB5R3	RAB35	PARK2	RBM39	RPA3	CTSB
ATM	OASL	RAB4A	ZRANB1	FAM20C	MCAM	INSR
GNAS	ITFG1	RAB5A	RAF1	DNAJC25	B3GNT2	FLT4
BAG6	BIRC3	RAB5C	MAP2K2	DNAJC5	MAP1LC3B	TYRO3
ZDHHC17	LMBR1L	RAB9A	FLT3	RNF128	NBR1	MET
CUL7	MEX3B	RPN1	PDK2	PMAIP1	CDC42	FGFR2
CCDC8	HIF1AN	RPN2	STK32C	BAG5	RAC1	RYK
EGFR	FANCD2	SEC62	ADCK3	ATG5	RHOG	CLEC4E
ILK	ORF50	STX4	POMK	ANKFY1	CCNF	TFRC
IRAK1	Nsp4ab	STX6	STK32B	ARF6	TRIM67	CLEC4D
TMEM129	PAK1	STX7	ORF3a	ATP2A1	CDK2	TMEM106B
CD3E	PRKAR1A	ZFPL1	ORF7a	B3GAT1	MOB2	TAX1BP1
HS2ST1	PXN	WDR5	IGF1R	C11orf52	TGOLN2	TOLLIP
HLA-DQA1	SHOC2	C7orf69	DDR2	CALR3	FURIN	KIF14
HLA-DPA1	VAV1	ASIC4	NTRK3	CAV1	IFITM1	NPC1
CD1B	EMC1	HLA-DQB1	FGFR1	CXADR	ANPEP	SCARA3
HLA-DRB5	EMC2	UGT1A7	PDGFRB	DIRAS3	CTSS	PRG2
ITM2B	EMC4	ADAM30	FGFR4	G3BP1	DPP4	RPS6KB2
IL20RB	MMGT1	DNASE1L1	ALK	IFITM3	BUD31	NTRK1
ERBB3	ORF8	HLA-DRB3	EPHA10	ACE2	GPR35	
GABRE	IMMP2L	BTNL2	ROR1	TMPRSS4	LTB4R2	

Table S2 The primer sequences used in the study

Gene	Forward primer (5′ to 3′)	Reverse primer (5′ to 3′)
OASL	CCATTGTGCCTGCCTACAGAG	CTTCAGCTTAGTTGGCCGATG
NBR1	GTGGGGCTTCATCAACGACA	AGATGGCAGTTAAACAGGGAAAC
18S	TGCGAGTACTCAACACCAACA	GCATATCTTCGGCCCACA
Oasl2	TTGTGCGGAGGATCAGGTACT	TGATGGTGTCGCAGTCTTTGA
Gapdh	AATGGATTTGGACGCATTGGT	TTTGCACTGGTACGTGTTGAT

Table S3 Antibodies information used in the study

REAGENT or RESOURCE	SOURCE	IDENTIFIER	Dilution
**Antibodies**			
OASL Rabbit mAb	zenbio	821065	WB:1:1000
OASL Polyclonal Antibody	Immunoway	YT6102	IHC:1:200
OASL Polyclonal Antibody	Invitrogen	PA5-72809	IF:1:400
OASL Polyclonal Antibody	abcam	ab191701	WB:1:1000
beta-actin antibody(HRP conjugated)	Sharebio	SB-AB2001	WB:1:3000
DYKDDDDK tag Polyclonal antibody (Binds to FLAG® tag epitope)	Proteintech	20543-1-AP	WB:1:1000<br>0IF:1:1000
Anti-HA Antibody(Mouse)	Yoche	AYC02-100	WB:1:5000<br>IF:1:200
HA-Tag (C29F4) Rabbit mAb	Cell Signaling Technology	3724S	WB:1:1000<br>0IF:1:1000
LAMP1 Antibody(1D4B)	Santa cruz	sc-19992	IF:1:100
LC3B (D11) XP® Rabbit mAb	Cell Signaling Technology	3868S	IF:1:2000
LC3B Antibody	Cell Signaling Technology	2775S	WB:1:1000
Anti-LC3B Antibody	abcam	ab192890	IHC:1:100
HLA class I ABC Polyclonal antibody	Proteintech	15240-1-AP	WB:1:5000<br>IHC:1:1000
Alexa Fluor® 647 anti-human HLA-A,B,C Antibody	Biolegend	311414	FC:1:200
ubiquitin Polyclonal antibody	Proteintech	10201-2-AP	WB:1:1000
ATG7 Rabbit Polyclonal Antibody	Immunoway	YN5670	WB:1:2000
NBR1 mouse Monoclonal Antibody(7C3)	Immunoway	YM3751	IHC:1:100
ATG5 (D5F5U) Rabbit mAb	Cell Signaling Technology	12994	WB:1:1000
SQSTM1/p62 (D5E2) Rabbit mAb	Cell Signaling Technology	8025	WB:1:1000
Beclin 1 Antibody	afantibody	AF300150	WB:1:1000
NBR1 Antibody(4BR)	Santa cruz	sc-130380	WB:1:1000
EEA1 Polyclonal antibody	Proteintech	28347-1-AP	IF:1:100
Anti -Ki67 Rabbit pAb	Servicebio	GB111141	IHC:1:500
Recombinant Anti-CD8 alpha Rabbit mAb	Servicebio	GB15068-100	IHC:1:400
PE/Cyanine7 anti-mouse CD8a Antibody	Biolegend	100722	FC:1:200
Anti-Granzyme B Mouse mAb	Servicebio	GB12093-100	IHC:1:500
APC anti-human/mouse Granzyme B Recombinant Antibody	Biolegend	372204	FC:1:200
Brilliant Violet 421™ anti-mouse IFN- γ	Biolegend	505830	FC:1:200

Antibody			
Brilliant Violet 605™ anti-mouse TNF-α Antibody	Biolegend	506329	FC:1:200
PE anti-mouse CD45.2 Antibody	Biolegend	109807	FC:1:400
Fixable Viability Stain 780 APC-CY7	Biolegend	557654	FC:1:1000
Alexa Fluor® 647 anti-mouse H-2Kb Antibody	Biolegend	116512	FC:1:200
Anti-rabbit IgG, HRP-linked Antibody	Cell Signaling Technology	7074	WB:1:3000
Anti-mouse IgG, HRP-linked Antibody	Cell Signaling Technology	7076P2	WB:1:3000
Polyclonal Goat Anti-Mouse IgG labeled by HRP	Servicebio	GB23301	IHC:1:500
Immunohistochemical specific goat anti-rabbit secondary antibod	Servicebio	G1213-100UL	IHC:1:200
Goat anti-mouse IgG H&L (Alexa Fluor® 647)	abcam	ab150115	IF:1:500
Donkey anti-rat IgG H&L (Alexa Fluor® 488)preadsorbed	abcam	ab150153	IF:1:500
Cy3 Labeled Goat Anti-Rabbit IgG	Servicebio	GB21303	IF:1:200
**Chemicals**			
DMSO	Sharebio	SB-CR012	
MG132	MedChemExpress	HY-13259	
CHX	Sigma-Aldrich	C7698	
BafA1	MedChemExpress	HY-100558	

Table S4 The Mass spectrometry results of proteins interacting with OASL

OASL IP			
Gene names	Scores	Coverage [%]	Peptides
OASL	6.6485	4.1	1
LAMP1	5.8312	2.6	1
CREG1	6.9589	8.2	1
FTL	11.995	17.7	2
CTSG	20	7.1	3
CAP1	6.1993	3.8	1
AZU1	6.2788	5.2	1
GM2A	6.7817	5.2	1
TUBB4B	19.788	34.4	13
PSMD2	12.8	3	2
PSMC2	12.688	5.8	2
